# Modulation of E-Cadherin Function through the AmotL2 Isoforms Promotes Ameboid Cell Invasion

**DOI:** 10.3390/cells12131682

**Published:** 2023-06-21

**Authors:** Aravindh Subramani, Weiyingqi Cui, Yuanyuan Zhang, Tomas Friman, Zhihai Zhao, Wenmao Huang, Pedro Fonseca, Weng-Onn Lui, Vani Narayanan, Justyna Bobrowska, Małgorzata Lekka, Jie Yan, Daniel E. Conway, Lars Holmgren

**Affiliations:** 1 Department of Oncology and Pathology, U2, Bioclinicum J6:20, Solnavägen 30 Karolinska Institutet, Solna, 171 64 Stockholm, Sweden; aravindhsubramani1987@gmail.com (A.S.); weiyingqi.cui@ki.se (W.C.); yuanyuan.zhang2@ki.se (Y.Z.); tomas.friman@pelago.se (T.F.); pedro.fonseca@ki.se (P.F.); weng-onn.lui@ki.se (W.-O.L.); 2 Department of Physics, Faculty of Science: 2 Science Drive 3, S7-01-10, Lower Kent Ridge Road, Singapore 117542, Singapore; phyzzh@nus.edu.sg (Z.Z.); phyhwen@nus.edu.sg (W.H.); phyyj@nus.edu.sg (J.Y.); 3 Mechanobiology Institute (MBI): T-Lab, #10-02, 5A Engineering Drive 1, National University of Singapore, Singapore 117411, Singapore; 4 Department of Biomedical Engineering, Virginia Commonwealth University, 401 West Main Street, Richmond, VA 23284, USA; narayananv@mymail.vcu.edu (V.N.); dconway@vcu.edu (D.E.C.); 5 Institute of Nuclear Physics, Polish Academy of Sciences, PL-31342 Krakow, Poland; justynabobrowska88@gmail.com (J.B.); malgorzata.lekka@ifj.edu.pl (M.L.)

**Keywords:** p60AmotL2, AmotL2, E-cadherin, actin, nuclear lamina, mechanotransduction, cancer, cancer invasion, metastasis

## Abstract

The spread of tumor cells and the formation of distant metastasis remain the main causes of mortality in cancer patients. However, the mechanisms governing the release of cells from micro-environmental constraints remain unclear. E-cadherin negatively controls the invasion of epithelial cells by maintaining cell–cell contacts. Furthermore, the inactivation of E-cadherin triggers invasion in vitro. However, the role of E-cadherin is complex, as metastasizing cells maintain E-cadherin expression, which appears to have a positive role in the survival of tumor cells. In this report, we present a novel mechanism delineating how E-cadherin function is modulated to promote invasion. We have previously shown that E-cadherin is associated with p100AmotL2, which is required for radial actin formation and the transmission of mechanical force. Here, we present evidence that p60AmotL2, which is expressed in invading tumor cells, binds to the p100AmotL2 isoform and uncouples the mechanical constraint of radial actin filaments. We show for the first time that the coupling of E-cadherin to the actin cytoskeleton via p100AmotL2 is directly connected to the nuclear membrane. The expression of p60AmotL2 inactivates this connection and alters the properties of the nuclear lamina, potentiating the invasion of cells into micropores of the extracellular matrix. In summary, we propose that the balance of the two AmotL2 isoforms is important in the modulation of E-cadherin function and that an imbalance of this axis promotes ameboid cell invasion.

## 1. Introduction

Mechanical force transmission via cell junctions significantly influences cell shape components, molding cells into structures consistent with proper organ function [1,2,3,4,5]. Cadherin transmembrane proteins are of particular interest due to their essential role in connecting cellular junctions to the intracellular cytoskeleton [6,7]. Comprehending how these biomechanical complexes orchestrate intrinsic and extrinsic forces is crucial for understanding the mechanisms driving cell coordination in multicellular organ formation.

During the progression towards malignancy, mechanical forces between cells restrict cellular mobility. Single or clusters of tumor cells must overcome these mechanical constraints to invade adjacent tissues and eventually metastasize to distant organs. Metastatic spread accounts for 90% of cancer-related mortality; thus, understanding the underlying invasion processes is vital for improving therapeutic design [8,9,10]. The concept of epithelial–mesenchymal transition (EMT) is strongly implicated in tumor cell invasion and metastasis [11,12,13]. Here, transcriptional activity activates a mesenchymal phenotype, leading to reduced E-cadherin expression and a subsequent loss of cell–cell contacts [14,15,16,17]. However, this concept is debated, as lineage tracing of mesenchymal and epithelial tumor cells in genetically engineered mice suggests that EMT may not be a requirement for metastasis [18,19]. Another argument challenging the concept of “complete” EMT is that some carcinomas and metastases retain E-cadherin expression [20,21]. This observation suggests the existence of a spectrum of intermediates between epithelial and mesenchymal phenotypes, referred to as epithelial–mesenchymal plasticity (EMP) [20,22,23,24,25,26]. Cells undergoing EMP may retain E-cadherin expression, but its function can be modulated by other mechanisms, such as internalization to intracellular compartments [27].

In this report, we explore a novel mechanism involving the regulation of E-cadherin activity and cellular invasion. We previously identified the Angiomotin (Amot, AmotL1, and AmotL2) family of scaffold proteins [28,29,30,31] and demonstrated that these proteins bind to distinct membrane receptors, thereby activating actin filament formation [32,33,34,35].

Amot proteins were also shown to be major regulators of the HIPPO pathway [36,37,38,39]. Amot proteins regulate the HIPPO pathway by controlling cytoskeletal actin, which, in turn, modulates the HIPPO effectors YAP/TAZ [40,41,42,43]. Additionally, Amot scaffold proteins directly bind YAP1 and interact with its negative regulator, the LATS1/2 kinases [43]. Consequently, Amot proteins influence YAP/TAZ regulation through various mechanisms. One Amot protein, AmotL2, comprises two isoforms—p100 and p60—encoded by separate promoters [34,44]. Notably, YAP1 transcribes the p100 isoform [45,46,47], while c-Fos activates p60AmotL2 [44]. Under normal conditions, both in vitro and in vivo, p100AmotL2 is expressed, while p60AmotL2 is activated by severe ischemia in vivo and severe hypoxia in vitro [44]. P100AmotL2 associates with E-cadherin in epithelial cells and localizes with VE-cadherin complexes in endothelial cells [33]. In both cell types, p100AmotL2 inactivation disrupts the association of cadherins with specific actin filaments that mechanically connect adjacent cells by running perpendicular to cellular junctions [33,34]. In vitro traction force microscopy experiments revealed that AmotL2 is necessary for transmitting mechanical force between endothelial cells. In vivo, endothelial-specific AmotL2 inactivation impairs aortic expansion during embryogenesis [34], while in epithelial cells, AmotL2 is essential for actomyosin contractility during blastocyst hatching [33].

In our previous research, we uncovered that p60AmotL2, expressed in invasive cancers, disrupts apico-basal polarity by sequestering intracellular polarity protein complexes [44]. In this study, we present evidence illustrating that p60AmotL2 exerts a dominant-negative effect by binding to p100AmotL2, subsequently inhibiting the formation of actin filaments connecting cellular junctions to the nuclear lamina. This interference results in ameboid migration, characterized by single cell invasion.

## 2. Results

### 2.1. The Expression of p60AmotL2 Results in the Sequestration of p100AmotL2 into Intracellular Vesicles

**Figure 1:**
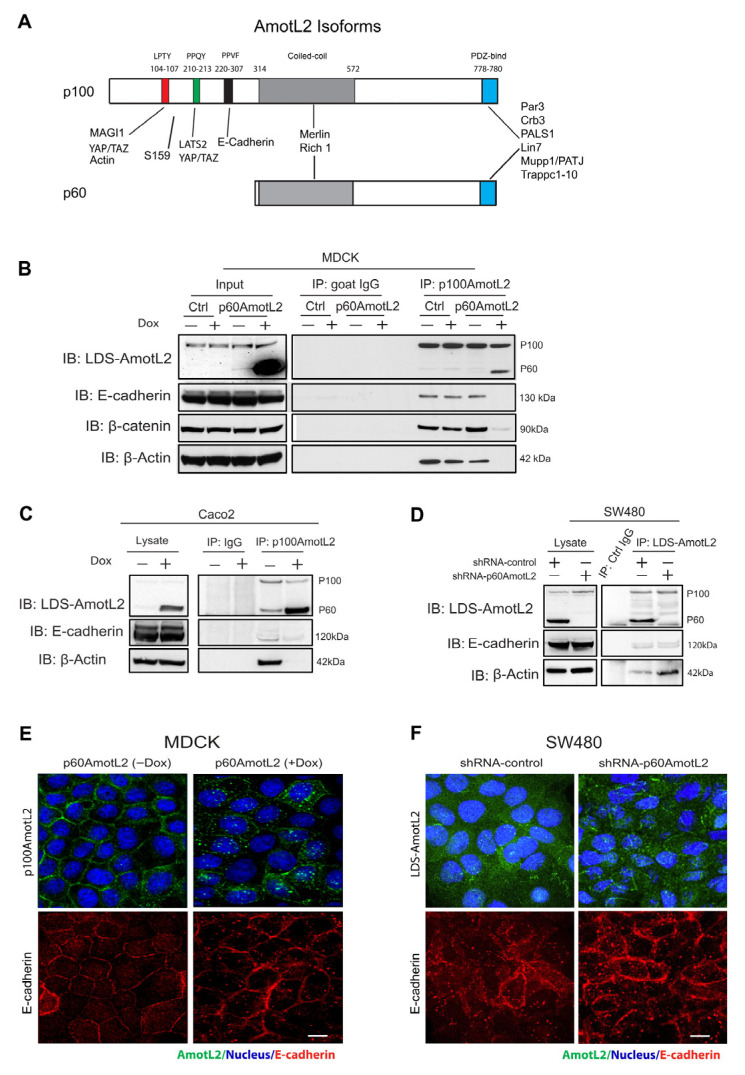
p60AmotL2 sequesters p100AmotL2 in intracellular vesicles. (**A**) Schematic figure illustrating the protein interactions of the two AmotL2 isoforms, p100 and p60AmotL2. The protein binding sites are indicated in the figure. The protein sequence is identical except for the N-terminal extension in the p100 isoform, which contains the E-cadherin binding site. The p100 and p60 isoforms also form heterodimers via binding to the coiled-coil domain (**B**) Left panel: Western blot analysis demonstrating the induction of p60AmotL2 in MDCK cells transfected with a Dox-inducible p60AmotL2 construct. The expression of p60 and p100AmotL2 was detected with antibodies that detect both isoforms (now depicted as LDS-AmotL2). Right panel displays immunoprecipitation of p100AmotL2 and subsequent detection of associated proteins. Immunoprecipitation was performed with p100AmotL2-specific antibodies, and Western blot was analyzed using LDS-AmotL2 antibodies. Note that p100AmotL2 binds to p60AmotL2 but not to E-cadherin, b-catenin and b-actin when p60AmotL2 iss induced. (**C**) Western blot analysis revealed lentiviral-mediated expression of p60AmotL2 in the Caco2 colorectal cancer cell line. Immunoprecipitation was performed with p100AmotL2-specific antibodies, and Western blot was analyzed using LDS-AmotL2 antibodies. (**D**) Western blot analysis indicating endogenous expression of p60AmotL2 in the SW480 colon cancer cell line and the specific shRNA knock-down of p60AmotL2, leaving p100 AmotL2 intact. Immunoprecipitation showed increase binding to b-actin after p60AmotL2 depletion (**E**) Immunofluorescent staining using p100 AmotL2-specific antibodies. Observe the delocalization of p100AmotL2 from cell–cell junctions to intracellular vesicles in p60AmotL2-expressing cells. (**F**) Immunofluorescent stainings of SW480 cells using LDS-AmotL2 antibodies. Note the localization of p100AmotL2 staining in cellular junctions after p60AmotL2 depletion. All experiments were repeated over three times. Size bars = 10 mm.

p100 and p60AmotL2 are identical in amino acid sequence, the exception being that the N-terminal domain of p100 contains PXPPY motifs that are responsible for interactions with actin, MAGI1b and YAP1 (Figure 1A). Additionally, there is an 87 a.a. sequence within the N-terminal domain that is also required for the interaction between p100AmotL2 and VE/E-cadherin [33,34]. The Amot family members may also form hetero or homo oligomers with each other, mediated by coiled-coil domains [29,32]. We hypothesized that p60AmotL2 might associate with p100AmotL2 and antagonize its normal function in a dominant-negative fashion. To test this hypothesis, we used Madin–Darby Canine Kidney cells (MDCK), commonly used for studies of cell–cell adhesion, polarity and the formation of 3-D structures in collagen. The MDCK cells were transfected with a doxycycline (Dox)-inducible p60AmotL2 construct (MDCK p60AmotL2), as previously described [44]. As shown in Figure 1B, the addition of Dox resulted in the induction of p60AmotL2 expression. We used an antibody targeting the N-terminal domain of p100AmotL2 for an immunoprecipitation analysis. The immunoprecipitation analysis using p100AmotL2-specific antibodies showed that p60AmotL2 and p100AmotL2 formed a complex. Furthermore, the induction of p60AmotL2 abrogated the association of p100AmotL2 to E-cadherin, β-catenin and actin (Figure 1B). The MDCK cells are immortalized but not transformed. We, therefore, assessed whether these data were valid also in human cancer cells. For this purpose, we transfected doxycycline-regulated p60AmotL2 in the colon cancer cell line Caco2. Caco2 cells maintain E-cadherin junctions and polarize when grown in 3-D matrices. As shown in Figure 1C, the p60AmotL2 expression inhibited the association of p100Amotl2 to both E-cadherin and actin.

The exogenous expression of a scaffold protein may sequester binding proteins in a non-physiological manner. To exclude this possibility, we analyzed the SW480 colon cancer cell line, which expresses both p100 and p60AmotL2 (Figure 1D). The endogenous and Dox-induced p60AmotL2 expression levels were comparable, as demonstrated in Figure S1B,C. We engineered a specific shRNA targeting the p60AmotL2 isoform, as depicted in Figure S1A. Interestingly, the depletion of p60AmotL2 resulted in the increased binding of p100AmotL2 to b-actin and, to a lesser degree, E-cadherin (Figure 1D and Figure S6C). Furthermore, we investigated the subcellular localization of p100AmotL2 and E-cadherin under two conditions: when exogenous p60AmotL2 was expressed and when endogenous protein was depleted. As shown in Figure 1E,F and Figure S1D,E, the expression of p60AmotL2 resulted in internalization of the p100AmotL2 isoform. Conversely, p60AmotL2 removal caused re-localization of p100AmotL2 to cell–cell junctions (Figure S1F–G).

### 2.2. p60AmotL2 Mirrors the Effect of p100AmotL2 Depletion

**Figure 2:**
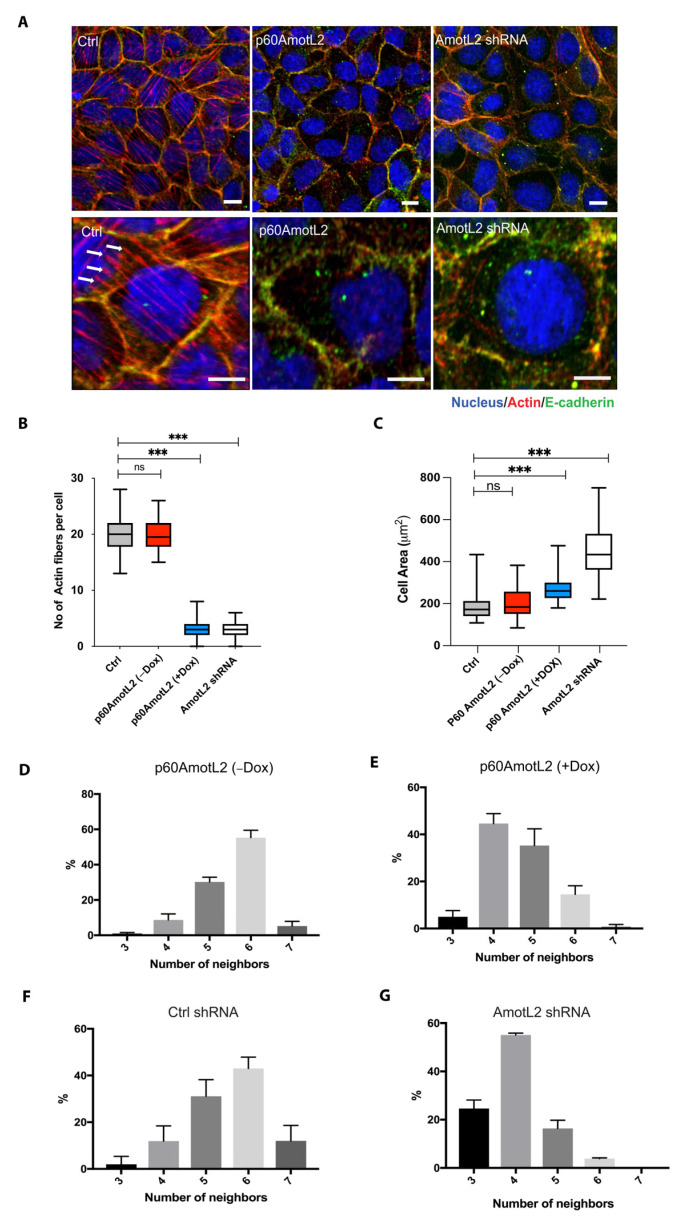
p60AmotL2 expression inhibits radial actin filaments similar to that of p100AmotL2 depletion. (**A**) Immunofluorescent staining of E-cadherin (green) and actin filaments visualized using phalloidin staining (red). Arrows indicate the presence of radial actin filaments in control MDCK cells, as shown by arrows in the higher magnification lower panel. These specific actin filaments were not detectable in p60AmotL2 or MDCK AmotL2 shRNA cells. (**B**) Box plot illustrating the quantification of radial actin filaments in MDCK cells (*n* = 50, Mann–Whitney U-test, *** *p* < 0.001). (**C**) Quantification of cell area (*n* = 50, Mann–Whitney U-test, *** *p* < 0.001). (**D**–**G**) Quantification of cell geometry, as analyzed by the number of neighboring cells (*n* = 50, Kolmogorov–Smirnov test, *p* < 0.001 on MDCK p60AmotL2 −Dox vs. +Dox and *p* < 0.001 on MDCK Ctrl vs. AmotL2 shRNA); The data are represented as mean ± SD, and all experiments were performed at least three times with similar results. *** *p* < 0.001. Scale bars = 20 µm.

We next evaluated whether the altered localization of p100AmotL2 influenced its ability to regulate radial actin filament formation. Immunofluorescent staining of the control MDCK cells displayed numerous junctional actin filaments that were orientated perpendicular to the cellular membrane (Figure 2A). Silencing p100AmotL2 with shRNA led to the loss of these junctional associated filaments, as we have previously reported [33,34,48]. Intriguingly, this was also observed in cells expressing p60AmotL2 (Figure 2A). Quantification of the phalloidin staining revealed that both silencing of p100AmotL2 and induced expression of p60AmotL2 had similar negative impacts on radial actin filament formation and cell area (Figure 2B,C).

We have previously demonstrated that the loss of p100AmotL2 not only affects the cytoskeleton but also disrupts cellular shape and epithelial packing [33]. The geometric packing of epithelia into hexagonal shapes is highly conserved across species. Depleting p100AmotL2 alters the shape of MDCK cells, causing them to become flattened, increase in cell area and lose hexagonal packing (Figure 2A,C). A similar effect on epithelial morphology was observed in cells expressing p60AmotL2 (Figure 2D–G). Collectively, these findings suggest that p60AmotL2 acts as an inhibitor of p100AmotL2 function.

### 2.3. p60AmotL2 Induces Single Cell Invasion in 3D Collagen

**Figure 3:**
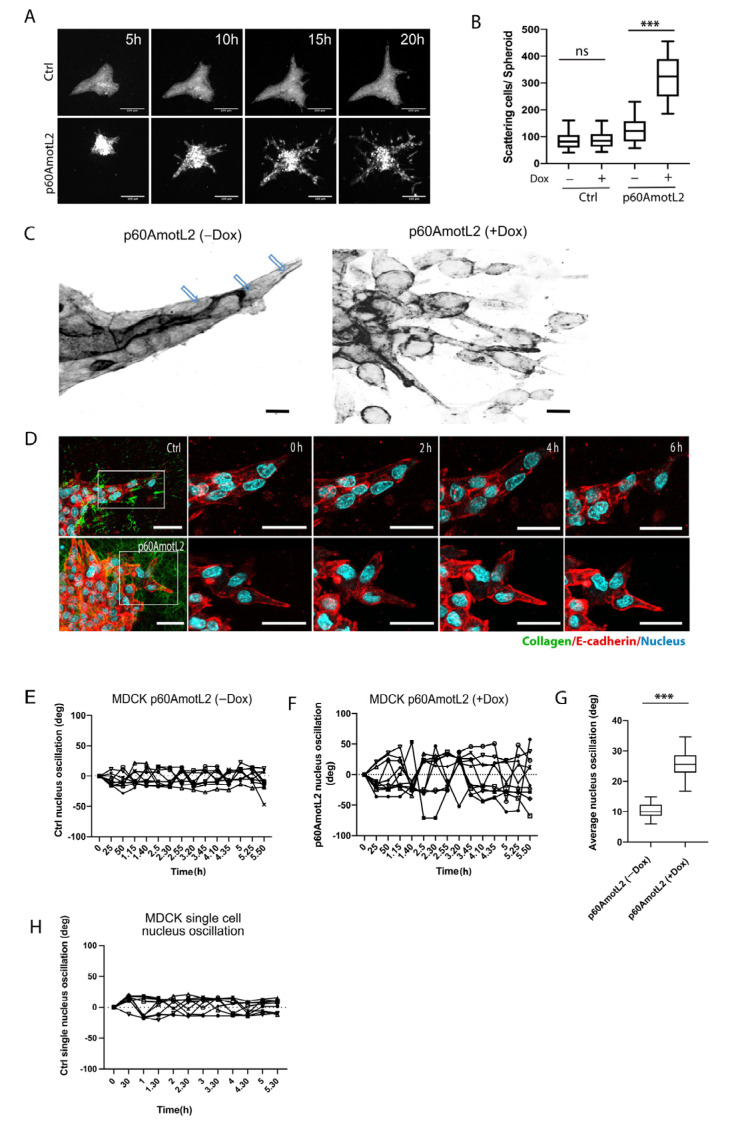
p60AmotL2 promotes cell scattering and invasion in an HGF-stimulated 3D collagen tubulogenesis assay. (**A**) Montage of time-lapse bright field movies of Ctrl MDCK cells (top panel) and p60AmotL2 (bottom panel) grown in collagen and stimulated with HGF. Size bars = 100 µm. (**B**) Quantification of cell scattering from the control and p60AmotL2-expressing cysts (*n* = 30, Mann–Whitney U-test, *** *p* < 0.001). (**C**) Inverted figures in black and white showing actin filaments in tube-forming sprouts of MDCK control cells and scattering cells of MDCK p60AmotL2 cells. Note the actin filaments that connect the leading cell to the following stalk cells in the control (as indicated by the arrows). (**D**) Live imaging of nuclear dynamics. Montage of time-lapse movies of MDCK control and MDCK p60AmotL2 cells. Actin was visualized using a SiR-Actin (red), and the nuclei were stained with Hoechst 33,342 (blue). Size bars = 50 mm. (**E**,**F**) The quantification of nuclear oscillations was made by calculating the degree of oscillation of the individual nuclei in the control and p60AmotL2-expressing cells using ImageJ. Each line shows the oscillation of the individual nuclei. (**G**) The box plots show the summary of oscillations of 30 nuclei from each group (*n* = 30, Mann–Whitney U-test, *** *p* < 0.001). (**H**) Shows the oscillation of the nuclei of single cells immersed in 3D Collagen. All experiments were performed three times with similar results, and the data are represented as mean ± SD.

MDCK cells naturally form cysts when embedded in collagen gels. When stimulated with hepatocyte growth factor (HGF), MDCK cells undergo branching morphogenesis in vitro [49] (Figure 3A top panel and Video S1). However, inhibiting E-cadherin promotes single cell invasion in the presence of HGF [50]. Consequently, we hypothesized that interfering with the E-cadherin/p100AmotL2 protein complex would impact in vitro migration and invasion. The MDCK cells were cultured without HGF in a collagen matrix for 10 days. Afterwards, cells were stimulated with HGF and imaged using time-lapse microscopy to observe the effects on cellular morphogenesis (Figure 3A). At 5 h, the control cysts initiated branching tubulogenesis, while p60AmotL2-expressing cells began to scatter, which dramatically increased over the next 20 h (Figure 3A bottom panel and Video S2).

During tubulogenesis, the control cells form sprouts with leader or leader cells connected to stalk cells, similar to the spatial coordination of endothelial cells observed during angiogenesis [51]. Immunofluorescent staining of the filamentous actin revealed the presence of long actin fibers connecting leader cells with stalk cells (Figure 3C and Figure S2). However, these longitudinal fibers were not detected in cellular buds emerging from p60AmotL2 cysts. We then used real-time microscopy to visualize the actin filaments and nuclear movements in p60AmotL2-expressing MDCK cells. The cells were labeled with SiR-actin dye and NucBlue to visualize the actin filaments and nuclei, respectively. Time-lapse imaging of the control cells showed that leader cell nuclei moved in the direction of the forming tube and that the nucleus elongated to align with the direction of collective migration (Figure 3D and Video S3). Interestingly, the nuclei of p60AmotL2-expressing cells appeared disconnected from the actin cytoskeleton, as nuclear oscillation was observed within the cells (Figure 3E–G and Video S4). In contrast, the single MDCK control cells grown in collagen did not exhibit a similar type of nuclear oscillation (Figure 3H). The same phenotypic changes were observed in adenoviral-p60AmotL2 infected cells (Figure S3).

**Figure 4:**
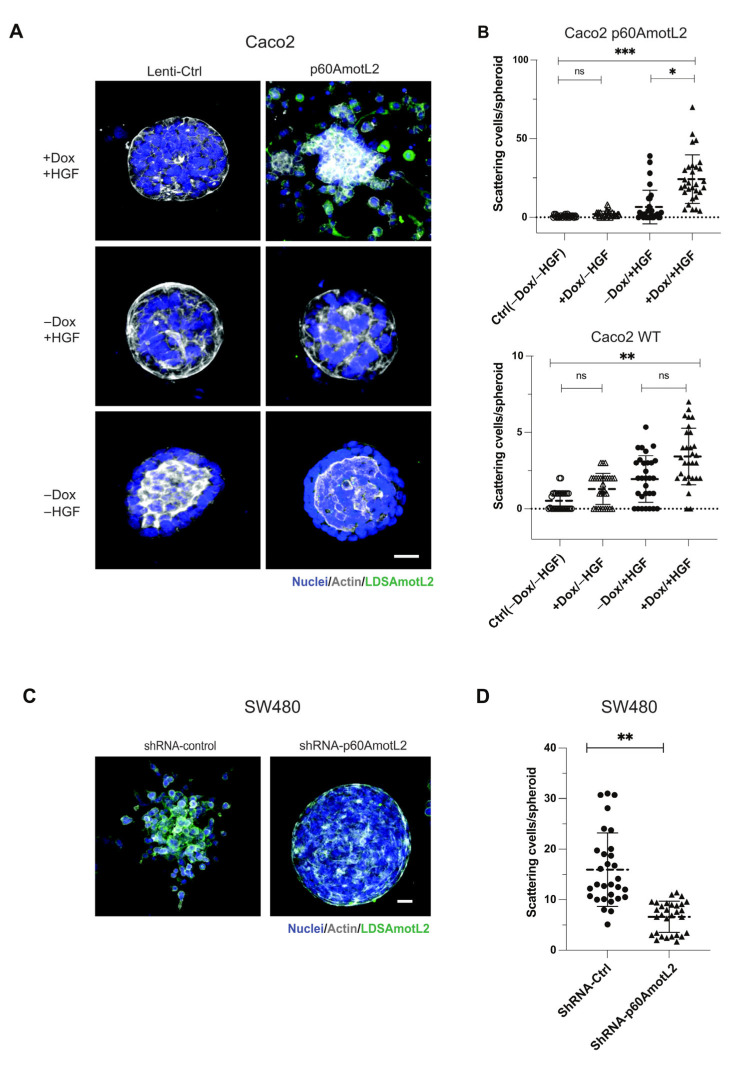
p60AmotL2 promotes cell invasion of colon cancer cell lines. (**A**) Caco2 cells were grown in 3D collagen for 7 days, after which control cells formed polarized cysts (**bottom panel**). Addition of Dox to Caco2 p60AmotL2 cells resulted in inhibition of the formation of polarized structures (**middle panel**). Addition of both Dox and HGF triggered single-cell invasion specifically in Caco2 p60AmotL2 cells (**top panel**). (**B**) Box plot diagram shows quantification of the cell invasion (*n* = 30, Mann–Whitney U-test, *** *p* < 0.001, ** *p* < 0.01, * *p* < 0.05). (**C**) SW480 Ctrl or shp60AmotL2-depleted cells were grown in 3D collagen. Note the single-cell invasion in Ctrl cells that was inhibited by the depletion of p60AmotL2. (**D**) Box plot shows the quantification of invasion in the experiment shown in C (*n* = 30, Mann–Whitney U-test, ** *p* < 0.01). All experiments were performed three times with similar results, and the data are represented as mean ± SD.

Caco-2 cells do not exhibit the same morphogenic response to HGF but form cysts when grown in a collagen matrix (Figure 4A,B). Interestingly, even in the absence of HGF, the induction of p60AmotL2 triggered cell scattering and single cell invasion from preformed cysts (Figure 4A,B). Conversely, SW480 cells spontaneously scattered when grown in collagen, which was hindered upon p60AmotL2 depletion (Figure 4C,D).

### 2.4. p60AmotL2 Negatively Affects Collagen Fiber Tension during 3-D Migration

**Figure 5:**
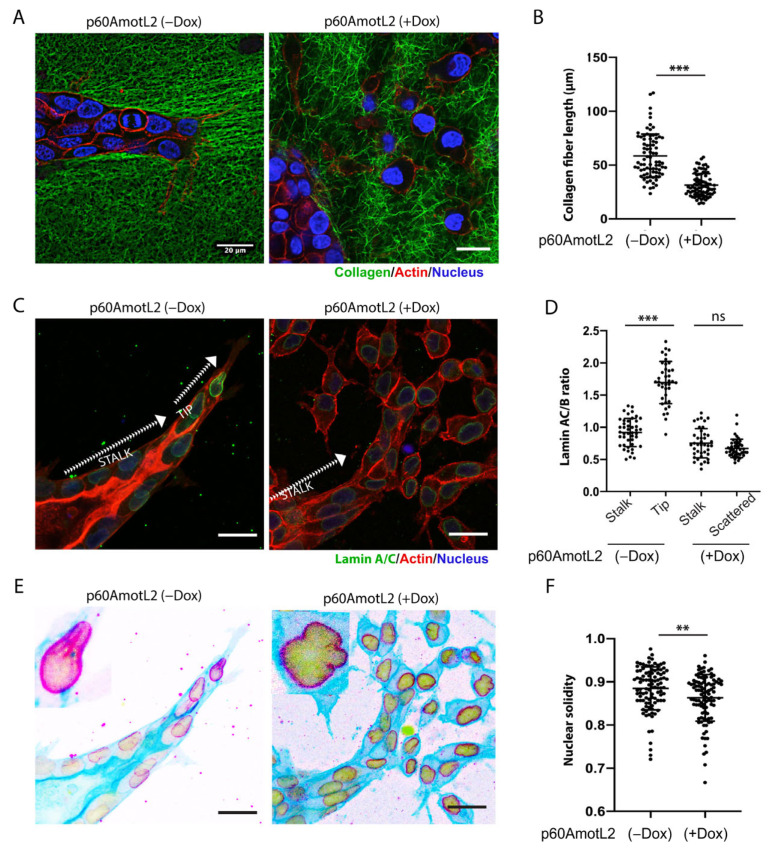
p60AmotL2 expression alters nuclear shape and integrity. (**A**,**B**) Collagen fibers were labeled with Oregon Green, actin with SiR-actin, and nuclei with NucBlue™ Live ReadyProbes™ Reagent. During the HGF-stimulated multicellular migration, the advancing sprouts interacted and pulled on the collagen fibers, something that was not detected in MDCK p60AmotL2 cells. (**B**) Fiber length was quantified as a measurement of cell–matrix interaction (*n* = 80, Mann–Whitney U-test, *** *p* < 0.001). (**C**) Immunofluorescent staining of Lamin A/C. Note the higher intensity staining of the leading cells in the controls. (**D**) The Lamin A/C to Lamin B ratio was quantified and presented in the dot plot (*n* = 40, Mann–Whitney U-test, *** *p* < 0.001). (**E**) The same picture as C but inverted to highlight the nuclear irregularities observed in p60AmotL2 cells. (**F**) Nuclear solidity was quantified using ImageJ (*n* = 102, Mann–Whitney U-test, ** *p* < 0.01). The data are represented as mean ± SD, and all experiments were performed three times with similar results. Size bars = 20 mm.

The extracellular matrix plays a vital role in cell migration by providing the tractional forces needed for forward movement. To examine the interaction between tube-forming and scattering cells within a 3D matrix, we labeled collagen with Oregon Green for the 3D culture. As is demonstrated in Figure 5A, the control MDCK cells elongated, and the sprouting cells interacted with the collagen, generating pulling forces that produced extended fibers aligned with the sprouting cells. In contrast, the p60AmotL2 cysts and scattered cells did not appear to deform the surrounding collagen in the same way (Figure 5A,B).

### 2.5. p60AmotL2 Affects the Integrity of the Nuclear Membrane

Nuclear lamins ensure structural integrity to the nuclear membrane. Lamin A (LMNA) is particularly interesting as it has been proposed as a “mechanostat” of mechanical forces. Matrix stiffness upregulates LMNA, thereby controlling the rigidity of the nuclear membrane [52]. The data in Figure 3 indicated that p60AmotL2 uncouples the nucleus from cellular actin filaments. Consequently, we sought to determine whether this uncoupling, induced by p60AmotL2, affected the properties of the nuclear lamina. We performed immunostaining of Lamin A in the HGF-treated control and p60AmotL2-expressing spheroids in collagen. In the protruding sprouts, it was evident that leader cells displayed a higher Lamin A/C signal compared to stalk cells (Figure 5C). However, no differences were observed between leader and stalk cells in p60AmotL2-expressing cysts. The Lamin A/C to Lamin B ratio was quantified and presented in Figure 5D and Supplementary Figure S3A. Defects in the LINC complex and regulation of LMNA were shown to reduce nuclear integrity and solidity [53]. We observed these associated defects in MDCK p60AmotL2 cells, where the nuclear membrane exhibited irregular contours (Figure 5E,F).

### 2.6. p60AmotL2 Detaches Radial Actin Filaments from the Nuclear Lamina

**Figure 6:**
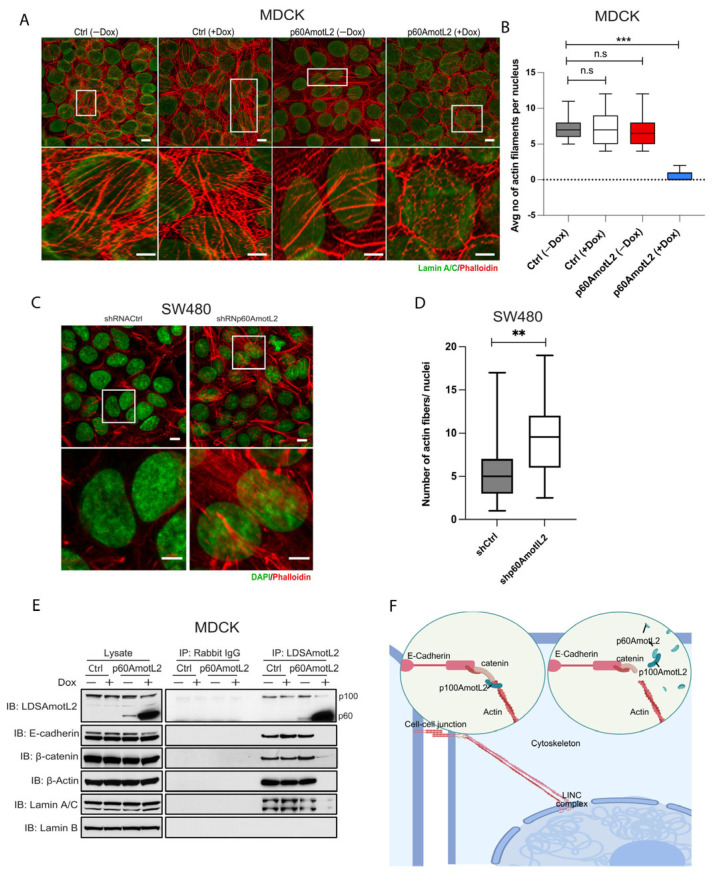
p60AmotL2 disrupts the connection of actin filaments to the nuclear lamina. (**A**). Immunofluorescence staining of the control and p60AmotL2-expressing MDCK cells. Phalloidin was used to visualize the actin filaments (Red), Lamin A/C (Green) and DAPI (Blue). The images were processed by ImageJ using a nanoJ-SRRF plugin. (**B**) Quantification of the number of actin filaments per nucleus in control and p60AmotL2-expressing cells (*n* = 50, Mann–Whitney U-test, *** *p* < 0.001). (**C**,**D**). Visualization and quantification of actin filaments in SW480 cells (*n* = 50, Mann–Whitney U-test, ** *p* < 0.01). (**E**) Co-Immunoprecipitation analysis showing the association of the E-cadherin/p100 AmotL2 complex to the LaminA/C is lost upon expression of p60AmotL2. (**F**) Schematic representation of mechanotransduction mediated by AmotL2 from cell–cell junctions to the nuclear lamina. All experiments were performed three times with similar results, and the data are represented as mean ± SD.

The nuclear lamina, composed of Lamin A/C and Lamin B, connects to the cytoskeleton through its association with the linker of nucleoskeleton and cytoskeleton (LINC) complex. The Klarsicht, ANC-1, Syne Homology (KASH) domain-containing protein, Nesprin-2, is situated in the outer nuclear membrane, where it directly interacts with actin filaments. We evaluated the nuclear lamina and cytoskeletal interactions using the immunofluorescent staining of actin and Lamin A/C (Figure 6A). The control cells displayed an abundance of actin filaments over the nuclear lamina, but this organization was disrupted by p60AmotL2 expression (Figure 6A, quantified in Figure 6B). This finding was confirmed by silencing endogenous p60AmotL2 in SW480 cells, which restored actin filaments associated with the nuclear lamina (Figure 6C,D).

As shown in Figure 1, E-cadherin and β-catenin were found to be associated with p100AmotL2. Following this observation, we proceeded to investigate if the LINC complex could be co-immunoprecipitated with p100AmotL2. Immunoprecipitation of p100AmotL2 revealed that Lamin A/C, but not Lamin B, was associated with this complex, and that p60AmotL2 expression abrogated this interaction (Figure 6E). Due to its large size (over 800 kDa), Nesprin-2 could not be detected. In summary, our data suggest a direct connection between E-cadherin in the outer plasma membrane and the nuclear lamina (Schematic illustrated in Figure 6F).

### 2.7. p60AmotL2 Impedes Force Transmission from Cell–Cell Junctions to the Nuclear Lamina

**Figure 7:**
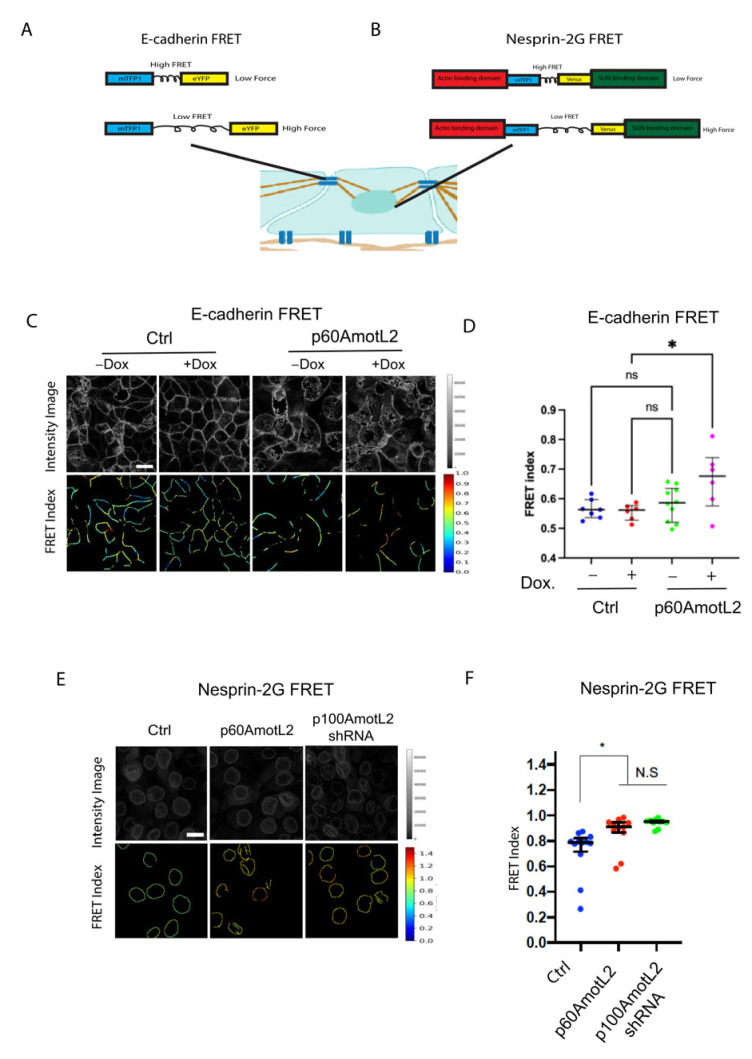
E-cadherin and Nesprin-2G tension sensors show reduced force in p60Amotl2-expressing cells. (**A**,**B**) Schematic showing the E-Cadherin and Nesprin-2G FRET constructs that were used. (**C**) The E-Cadherin FRET construct localized to the cellular junctions in transfected MDCK cells. FRET index is shown in the upper panel. The E-cadherin tension sensor had significantly higher FRET (indicating lower force) in +Dox p60AmotL2 cells (extreme **right panel**). (**D**) The box plot shows FRET index measurements across MDCK Ctrl and MDCK p60AmotL2 cells, depicting a significantly lower force (higher FRET) across E-cadherin in p60AmotL2 expressing cells. The midline represents the median of the estimates, the extent of the box represents the IQR, the whiskers denote the largest observation within 1.5xIQR, and outliers exceeding this are indicated by “+” (representative data from three independent experiments are shown, * *p* < 0.05, Student *t*-test). (**E**) The FRET index and fluorescence intensity of NespTS cells and p60AmotL2 cells were shown in the top and **bottom panel**. (**F**) The box plot shows FRET index measurements across the nuclei of the control and p60AmotL2 and shp100AmotL2cells, depicting a significantly lower force (higher FRET) across Nesprin-2 and in the MDCK p60AmotL2 cells as compared to the controls. The midline represents the median of the estimates, the extent of the box represents the IQR, the whiskers denote the largest observation within 1.5xIQR, and outliers exceeding this are indicated by “+” (representative data from three independent experiments are shown, * *p* < 0.05, Student *t*-test).

Figure 6F presents a hypothetical model suggesting that mechanical force may be transmitted from cell–cell junctions to the nuclear membrane through the p100AmotL2 protein complex. Additionally, this model proposes that p60AmotL2 disrupts the transmission of mechanosignals between cell–cell junctions and the nucleus. To examine this further, we measured the forces exerted on cellular junctions and the nucleus using fluorescence resonance energy transfer (FRET)-based biosensors for E-cadherin and Nesprin-2 (Figure 7A,B). When force is applied to these biosensors, two fluorescent tags (mTFP1 and EYFP/Venus) are displaced from each other, resulting in a change in FRET. Our findings indicate that both p60AmotL2-expressing cells and p100AmotL2-depleted cells display a higher FRET index (lower force) for the E-cadherin and Nesprin-2 biosensors (Figure 7C–F).

**Figure 8:**
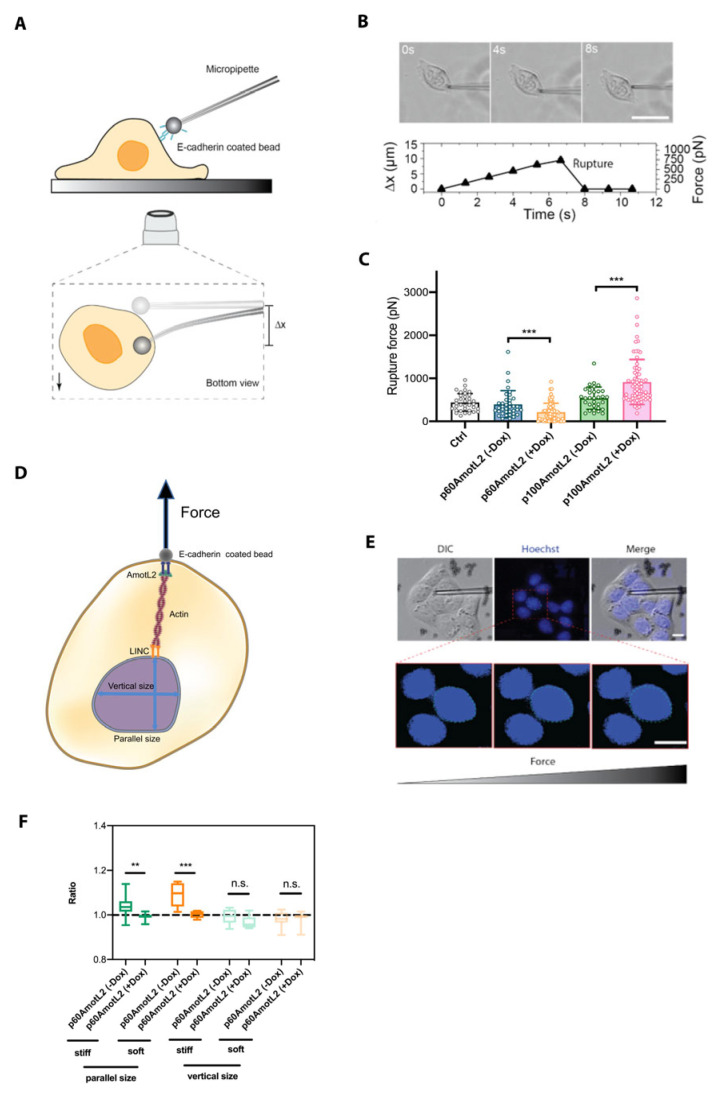
E-cadherin-coated micropipette force probe shows lowered cell–cell junctional stability in p60AmotL2 cells. (**A**) Schematic overview of micropipette force probe: The tip of the flexible micropipette holds the E-cadherin-coated bead through liquid aspiration pressure. The bottom view represents the bead displacement (Δx) when the cell is in contact with the micropipette. (**B**) Corresponding brightfield images and graph showing the micropipette deflection tracked over time until the adhesion interface ruptures. Size bar = 40 mm (**C**) Evaluation of the amount of force applied on the adhesion interface based on the micropipette deflection distance (Δx) and pre-calibrated bending stiffness (k) of the micropipette (*F* = *k*Δ*x*) (*n* > 30, unpaired two-sided Student *t*-test, *** *p* < 0.001). (**D**) Schematic showing the nuclear diameter measurement along the parallel and vertical direction of the E-cadherin-coated bead pulling. (**E**) Corresponding differential interference contrast (DIC) images together with Hoechst staining to monitor the diameter change of the nucleus, tracked over time. Size bars = 10 mm (**F**) The results demonstrate a significant change in parallel size following the induction of p60AmotL2 on both soft and stiff matrices as compared to control (*n* > 10, unpaired two-sided Student *t*-test, *** *p* < 0.001, ** *p* < 0.01). All experiments were performed three times with similar results, and the data are presented as mean ± SD.

To further investigate the mechanobiological property of the E-cadherin connection to the nucleus, we used a micropipette force probe (MFP) with a known bending stiffness (k). MFP employs a calibrated hollow glass rod holding a 3 μm bead coated with E-cadherin at the tip, as illustrated in Figure 8A. The bead is next guided into contact with the cell. At this stage, force is exerted onto the bead via the micropipette, which, in turn, induces a bending of the pipette. This continues until the point of rupture of the interaction between the bead and the cell. (Figure 8B). The bead displacement is tracked and recorded using an inverted microscope, and the corresponding forces are evaluated as shown in Figure 8C. The results indicate that p60AmotL2 induction lowers E-cadherin junctional rupture force, while p100AmotL2 overexpression increases E-cadherin junctional stability (Figure 8C).

Since we hypothesize that p100AmotL2 is involved in linking E-cadherin to the nucleus through radial actin filaments, we questioned whether pulling on E-cadherin might affect the nuclear shape and movement. To assess nuclear displacement or size change during E-cadherin-coated bead pulling, we cultured cells on both stiff (glass) and soft (polydimethylsiloxane (PDMS)-fibronectin) substrates. The nuclear size change ratios were calculated by comparing the initial size before pulling, both parallel and perpendicular to the force applied on the nucleus by MFP (Figure 8D). The findings demonstrate that p60AmotL2 induction significantly alters the size of the nucleus in the direction parallel to MFP pulling when cells are cultured on either soft or stiff substrates (Figure 8D–F).

### 2.8. p60AmotL2 Modifies the Physical Properties of the Nuclear Membrane

**Figure 9:**
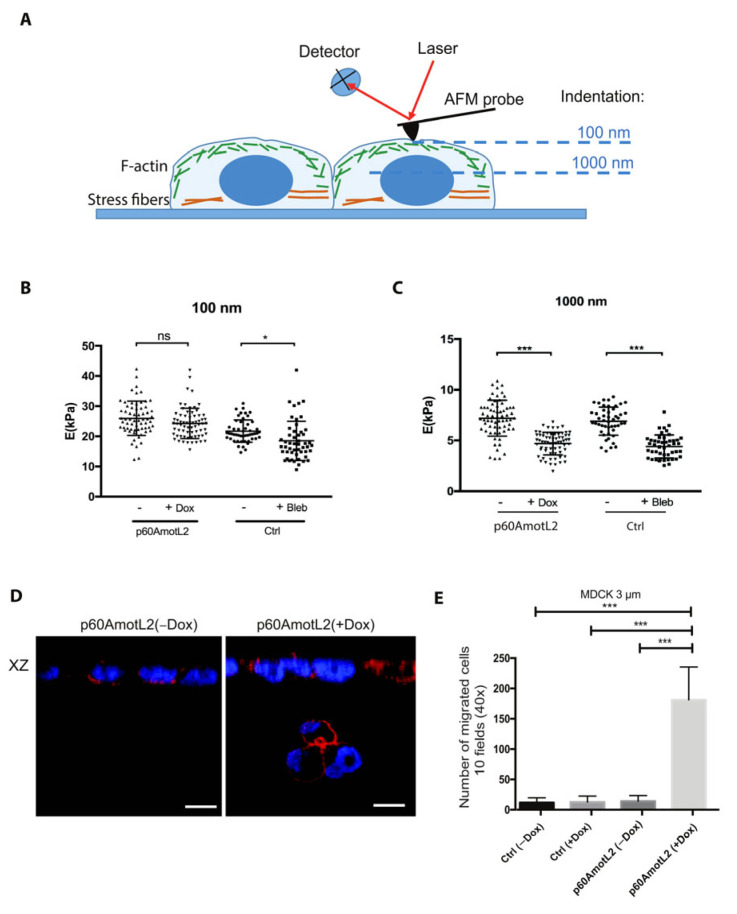
p60AmotL2 expression promotes nuclear plasticity and invasion. (**A**) Schematic representation of Atomic force microscopy. Measurements were made at 100 nm and 1000 nm in order to analyze the properties at the cell cortex and nucleus, respectively. (**B**,**C**) Quantification of Young’s modulus E (kPa) in the MDCK cells at 100 and 1000 nm as indicated. Induction of p60AmotL2 significantly reduced the Young’s modulus at 1000 nm, reflecting an increased plasticity of the cell nucleus (*n* > 50, unpaired two-sided Student’s *t*-test, *** *p* < 0.001, * *p* < 0.05). (**D**) XZ axis shown of cells stained with phalloidin (red) and DAPI (blue). The cells were seeded on a 3 μm Transwell filter in order to analyze the ability of cells to migrate through a confined environment. Note the p60AmotL2 cells that have migrated through the pores of the transwell filter (arrow). Size bars = 20 μm. (**E**) Bar diagram depicts the number of cells that have migrated through the 3 μm pore Transwell filter (Mann–Whitney U-test, *** *p* < 0.001). All experiments were performed three times with similar results and the data are represented as mean ± SD.

We next tested whether mechanical forces applied to the nuclear membrane would alter its physical properties in the presence of p60AmotL2. Therefore, we employed atomic force microscopy (AFM) to analyze the nuclear stiffness (Figure 9A). Here, a cantilever (a delicate flat spring) indents the cell in situ, and nuclear stiffness can be determined indirectly by analyzing large indentation depths, in this case, 1000 nm. In AFM, the deflection of the cantilever measured during cell indentation is compared with that recorded on a stiff substrate (e.g., Petri dish surface) and converted into Young’s Modulus using the Hertz–Sneddon mechanical model [54].

However, at large indentations, the mechanical response of the cells to the applied load forces also includes contributions from various organelles within the cell, including the nucleus. Since the nucleus is by far the largest organelle of the cell, we hypothesized that its contribution to Young’s modulus is the most significant.

Our analysis revealed that the MDCK cells induced to express p60AmotL2 exhibited a stiffness comparable to the control cells for small indentations (100 nm) but significantly lower nuclear stiffness for larger indentations (1000 nm). The latter was similar to cells treated with the actomyosin inhibitor blebbistatin (Figure 9C).

### 2.9. p60AmotL2 Hinders the Transmission of Force from Cell–Cell Junctions to the Nuclear Lamina

We then investigated whether the increased deformability of p60AmotL2-expressing cells also corresponded to an enhanced capacity to migrate through micropores. Using 8 μm pore transwells, we demonstrated that p60AmotL2-expressing cells had a higher propensity to migrate than control cells (Figure S4A,B). However, a large majority of the control cells also exhibited migration through the 8 μm pores (Figure S4B). Subsequently, we decreased the pore size to 3 μm, which resulted in inhibition of the migration of the control cells through the filter. In contrast, the migration of the MDCK p60AmotL2 cells was still detected, supporting the hypothesis that the size of the nucleus constrains its ability to pass through filter pores, as demonstrated in Figure 9D,E. The expression of p60AmotL2 led to over 150 migrating cells/field, and the nuclei of translocating cells displayed an altered “hourglass”-like morphology (Figure 9D,E). These findings were replicated in Caco2 cells, and the migration of SW480 cells was inhibited after p60AmotL2 depletion (Figure S4C–F).

## 3. Discussion

The loss of E-cadherin is strongly associated with EMT and metastasis formation, where deregulation of cell–cell adhesion was demonstrated to trigger cancer-cell dissemination from the primary tumor [55,56,57]. However, the significance of E-cadherin in invasion was recently debated, as many tumors and corresponding metastases retain E-cadherin expression [21]. Moreover, it was shown that E-cadherin-positive tumor cells possess a higher ability to form lung metastases than their negative counterparts in vivo [18]. This suggests that E-cadherin expression is dynamic and reversible, pointing to the influence of alternative mechanisms impacting expression levels.

In this study, we demonstrate for the first time that E-cadherin function may be modulated by the relative expression of the two AmotL2 isoforms. The p100AmotL2 isoform is ubiquitously expressed in epithelial cells and associates with E-cadherin [33]. We have previously shown that the association of p100AmotL2 with E-cadherin or VE-cadherin in endothelial cells triggers radial actin filament formation, which transmits mechanical force between neighboring cells [33,34]. In this study, we show that in epithelial cells, these actin filaments connect to the nuclear lamina, providing a direct link between cell–cell junctions and the nucleus. Moreover, these forces could be propagated to larger cell populations rather than single cells, suggesting that mechanical forces can coordinate cellular functions at supra-cellular levels [58]. Our previous findings indicate that p100AmotL2 is required for blastocyst hatching and the expansion of the developing aorta [33,34]. It is, thus, tempting to speculate that p100AmotL2 can confer similar long-distance coordination of force via cell–cell junctions to regulate these developmental processes.

Epithelial layers separate internal structures from external environments, such as in the gut, where the intestinal lumen is separated from the rest of the body by an epithelial barrier [59,60]. These epithelial barriers form a tight monolayer, preventing cell delamination and barrier integrity loss. However, cells may be shed from this layer via apical extrusion to maintain tissue homeostasis [61]. The Amot proteins form oligomers via binding of the coiled-coil domain [29]. The expression of p60AmotL2 may interfere with the tight coupling between epithelial cells, as we show that p60AmotL2 binds and relocates the p100 isoform to intracellular vesicles. This phenocopies the cellular effects of p100AmotL2 depletion and positions p60AmotL2 as a dominant-negative regulator of p100AmotL2. Interestingly, p60AmotL2 also uncouples the actin filaments binding to the nuclear membrane. The properties of the nuclear lamina were recently shown to be crucial in regulating single-cell migration [62,63,64,65].

Reducing LMNA levels results in increased nuclear membrane elasticity, facilitating migration through micropores in the ECM [66,67]. During tubulogenesis, we observed that the leading tip cells of collectively migrating cells displayed elevated Lamin A/C expression. However, this pattern of expression was absent in budding tip cells of p60AmotL2-expressing cysts. This suggests that disconnecting the nuclear cytoskeleton from the nuclear lamina inhibits LMNA expression modulation in response to force stimuli. However, we did not examine the phosphorylation status of Lamin A/C, which was shown to control the physical properties of the nuclear lamina [68].

Interestingly, the p60AmotL2 isoform is not typically detected under in vitro culture conditions; however, it exhibits high expression in various invasive cancer types, including colorectal and cervical cancers. Its expression is correlated with poor prognosis in these malignancies [44]. Given that p60AmotL2 is induced by severe hypoxia, we propose that this isoform is regulated by micro-environmental factors in a reversible manner. The normal function of p60AmotL2 remains unclear, and further studies are needed to determine its potential importance, for instance, in the apical extrusion of cells in the gut epithelium. Importantly, it will be of interest to investigate whether p60AmotL2 expression confers increased resistance or sensitivity to therapy.

## 4. Material and Methods

### 4.1. Antibodies

The following primary antibodies were used: LDS AmotL2 (polyclonal antibodies reactive to human AmotL2 C-terminal peptide and detecting both p60 and p100 isoforms [34], p100-specific N-terminal of AmotL2 (product no. sc-82501, Santa Cruz Biotechnology, Santa Cruz, CA, USA), β-catenin (product no. 610154, BD Biosciences, Franklin Lakes, NJ, USA), E-cadherin (product no. 610193, BD Biosciences), actin (product no. ab3280, Abcam), Lamin A/C (product no. ab108922, Abcam, Cambridge, UK), Lamin B (product no. ab220797, Abcam) and Nesprin-2G (product no. MABC86, Sigma-Aldrich, St. Louis, MO, USA). Positive staining was visualized using the following secondary antibodies: Alexa 488 anti-rabbit (product no. ab150077, Abcam), Alexa 568 anti-mouse (product no. A-11004, Invitrogen, Waltham, MA, USA), ECL anti-mouse IgG horseradish peroxidase (product no. NA931V, Sigma-Aldrich) and ECL anti-rabbit IgG horseradish peroxidase (product no. NA934V, Sigma-Adrich). The following control IgGs were used in co-immunoprecipitation experiments: IgG from rabbit serum (product no. I8140, Sigma-Aldrich), IgG from mouse serum (product no. I8765; Sigma-Aldrich) and IgG from goat serum (product no. I5256, Sigma-Aldrich). The actin filaments were visualized using Texas Red (product no. T7471, Thermo Fisher Scientific, Waltham, MA, USA) or Atto 647 phalloidin (product no. 65906, Sigma-Aldrich). The nuclei were counterstained using 4’,6-Diamidine-2’-phenylindole dihydrochloride (DAPI) (product no. F6057, Sigma-Adrich). For live cell imaging, the nuclei were visualized using NucBlue™ Live ReadyProbes™ Reagent (product no. R37605, Thermo Fisher Scientific). The actin was stained using SiR-actin Kit (product no. CY-SC001, Spirochrome, Stein Am Rhein, Switzerland).

### 4.2. Cell Culture

Madin-Darby Canine Kidney (MDCK) (PTA-6500, American Type Culture Collection, Manassas, VA, USA), Caco2 (product no. 86010202, Sigma-Aldrich) and SW480 (product no. 87092801, ECACC, Porton Down, UK) cells were cultured in Dulbecco’s Modified Eagle Medium (DMEM) (product no. D6429, Sigma-Aldrich) supplemented with 10% fetal bovine serum (FBS, product no. 10270106, Thermo Fisher Scientific) and penicillin/streptomycin (product no. 15070063, Thermo Fisher Scientific). Both the MDCK and Caco2 cell lines stably expressing doxycycline-inducible p60AmotL2 were produced with the Gateway™ system (Thermo Fisher Scientific), as previously described [44]. The MDCK AmotL2 shRNA cells were generated using lentiviral vectors as described [33]. The exon 9 of the AMOTL2 encoding mRNA has tandem splicing acceptor sites at the 5′ end of exon 9, where the splice sites are located 3 bases from each other. The splicing for the exon 9 of AMOTL2 p100 occurs at the proximal splice site, while the AMOTL2 p60 occurs at the distal splice site. To specifically deplete cells of p60AmotL2, we designed an shRNA to target the sequence spanning the exon 8 and exon 9 junction of AMOTL2 p60, as illustrated in Figure S1A. Two oligonucleotides (5′CCGGTGCTGACTACAGACAGAGCACCTCGAGGTGCTCTGTCTGTAGTCAGTTTTTG and 5′-AATTCAAAAACTGACTACAGACAGAGCACCTCGAGGTGCTCTGTCTGTAGTCAGCA) were annealed and cloned into the AgeI and EcoRI sites of pLV[shRNA]-Puro-U6 (VectorBuilder Inc., Chicago, IL, USA)”.

### 4.3. Western Blot (WB)

For lysate preparation, the cells were treated with a lysis buffer that contained 50 mM Hepes buffer, 150 mM NaCl, 1.5 mM MgCl_2_, 1 mM EGTA, 10% glycerol and 1% TritonX-100, along with a freshly added 1X protease inhibitor (product no. 04693159001, Roche, Basel, Switzerland). This process was performed on ice. Following this, the mixture was subjected to centrifugation at 15,000 rpm for a span of 3 min and supernatant was collected. Lysates were then combined with SDS sample buffer (4X, product no. 1225644, Novex, Wadsworth, OH, USA), which was enhanced with a 10% sample reducing agent (product no. 1176192, Novex). The proteins present were fractionated using a Bis-Tris precast polyacrylamide gel with a gradient of 4–12% (product no. NP0322BOX, Novex). These fractionated proteins were then moved onto a nitrocellulose membrane (product no. 10401396, Whatman, Maidstone, UK). To block any non-specific binding, the membrane was treated with a solution of 5% non-fat milk and 0.1% Tween 20 in PBS for an hour at room temperature. Following this, the membrane was incubated overnight at 4 °C with the primary antibody and was then subjected to treatment with the secondary antibody for an additional hour at room temperature. Finally, the proteins that were labeled with antibodies were identified using a chemiluminescent substrate (ECL; product no. RPN2232, Amersham plc., Amersham, UK).

### 4.4. Co-Immunoprecipitation (co-IP)

For the co-immunoprecipitation (co-IP) experiment, the cells were rinsed with ice-cold PBS and directly scraped off a 10 cm dish into a lysis buffer. The lysis buffer consisted of 50 mM Hepes buffer, 150 mM NaCl, 1.5 mM MgCl_2_, 1 mM EGTA, 10% glycerol and 1% TritonX-100. The lysates were then centrifuged at 15,000 rpm for 5 min to remove debris. To minimize non-specific binding to Sepharose beads, the cell lysates were pre-cleared by incubating with protein G Sepharose beads (product no. ab193259, Abcam, Cambridge, UK) for 1.5 h at 4 °C. After pre-clearing, 2 µg of AmotL2 or control antibodies from the same species were added to the lysates and incubated overnight at 4 °C. The immunocomplexes were precipitated by adding protein G beads to the lysate and incubating for 2 h at 4 °C. The beads were then washed five times by centrifugation at 12,000 rpm for 20 s each time with the lysis buffer to remove non-specifically bound proteins. Finally, the protein–bead complexes were denatured, and the proteins were separated from the beads for analysis by Western blotting.

### 4.5. Immunofluorescence Staining

The cells were grown on chamber slides for the immunofluorescence analysis (product no. 177402, Thermo Fisher Scientific). The slides underwent a one-minute wash in PBS, after which they were fixed in 4% paraformaldehyde (PFA; product no. sc-281692, Santa Cruz Biotechnology) in PBS for a duration of 10 min. The cells were then washed three times for five minutes each in PBS and blocked for an hour in 5% horse serum (product no. 16050, Thermo Fisher Scientific) in PBS. Following this, the cells were incubated with the respective primary antibody for an hour in 5% horse serum in PBS. The slides were then washed three times for five minutes each in PBS, after which they were treated with the respective secondary antibody in 5% horse serum in PBS. After further washing with PBS (three times for five minutes each), the slides were prepared for microscopy with Fluoroshield™ containing DAPI (product no. F6057, Sigma-Aldrich). The images were subsequently acquired using a Zeiss LSM 700 confocal microscope (Oberkochen, Germany).

### 4.6. Three-Dimensional (3-D) Cultures in Collagen Gels

The cells in the proliferative phase were collected and incorporated into collagen gels. The gel matrix was composed of PureCol^®^ bovine collagen (2 mg/mL, product no. 5005, Advanced BioMatrix, Carlsbad, CA, USA), M199 media (1x, product no. M0650, Sigma-Aldrich), sodium bicarbonate (NaHCO_3_, 25 mM, product no. 25080094, Thermo Fisher Scientific), and FBS (10%, product no. 10270106. Thermo Fisher Scientific). The cells were seeded into the gel at a concentration of 5000 cells per mL of gel. The medium was replaced on the day following cell seeding and, subsequently, every three days. Doxycycline (10 ng/mL, product no. D3447, Sigma-Aldrich) was added to the culture seven days post-seeding. Growth factors and/or inhibitors treatment commenced on day ten. The experiments were terminated on either day 11 or 12.

Alternatively, doxycycline was introduced into the culture the day after cell seeding. Three-dimensional (3-D) cultures were sustained with or without doxycycline for a period of up to two weeks.

### 4.7. Immunofluorescence Staining of 3-D Gels

The collagen gels underwent two washes in PBS and were then fixed in 0.5 mL of 4% PFA (product no. sc-281692, Santa Cruz Biotechnology) for half an hour. Following this, the gels were washed three times with PBS and permeabilized using 0.1% Triton X-100 (product no. X100, Sigma-Aldrich) for a duration of 15 min. After an additional two washes in PBS, the primary antibody, diluted in 5% normal horse serum, was added and allowed to incubate at 4 °C overnight with gentle shaking. The gels were subsequently washed three more times in PBS and then incubated with the secondary antibody for 1.5 h at room temperature, again with gentle shaking. Following another three washes in PBS, the gels were prepared for imaging with ProLong™ Gold Antifade Mountant (product no. P36930, Thermo Fisher Scientific). Confocal images were captured using a Zeiss LSM 700 microscope (Oberkochen, Germany), and all image processing was carried out using ImageJ software.

### 4.8. Live Cell Imaging

The MDCK wild-type cells or MDCK cells expressing p60AmotL2 were integrated into collagen gels and cultured, as detailed previously, until day 10. For certain gels, the collagen matrix was fortified with collagen conjugated with Oregon Green (product no. O10241, Thermo Fisher Scientific) at a final concentration of 30 µg/mL. The gels with matured cysts were first stained with CellMask™ Orange (product no. C10045, Thermo Fisher Scientific), a cell membrane dye. This involved adding 5 µg/mL of CellMask™ Orange in Hank’s Balanced Salt Solution (HBSS; product no. 14025092, Thermo Fisher Scientific) to each gel and incubating for an hour at 37 °C.

Subsequently, the gels were washed once in HBSS, and the cells were treated with phenol red growth media for another hour. The treatment media was then added, and after 4–5 h, the chamber slides holding the gels were mounted on a Zeiss LSM 700 microscope (Oberkochen, Germany) that was equipped with a humidified incubator chamber (5% CO_2_ and 37 °C). The time-lapse confocal stacks were captured and shown as either movies or images derived from these movies, where each frame constitutes a maximum image intensity projection. The cell scattering in 3-D assays was scrutinized through both fluorescent and time-lapse imaging. Both the fluorescent and time-lapse images were inspected and quantified using IMARIS software (https://imaris.oxinst.com (accessed on 14 January 2019)). Clusters comprising fewer than three cells were categorized as scattered.

### 4.9. Analysis of Nuclear Oscillation

Nuclear oscillation was examined employing a time series method with the aid of ImageJ software. The X and Y coordinates of each nucleus were derived from time-lapse images, enabling the portrayal of nuclear movement over time. The oscillation angle was ascertained by gauging the maximum displacement of the nucleus from its initial position across the time series. This measurement was achieved by utilizing the “line tool” function available in ImageJ. To facilitate the quantification of these measurements, the “angle” option within the “Set Measure” feature was activated. This allowed for a detailed analysis of the degree and direction of nuclear oscillation over the course of the experiment.

### 4.10. Analysis of Lamin A/C to Lamin B Ratio

The Lamin A/C to Lamin B ratio was determined through processing the fluorescent images immunostained for both Lamin A/C and Lamin B (in separate channels). These images underwent processing in ImageJ by first carrying out maximum intensity projections for each image and then converting them into grayscale. The image thresholds for each channel were independently adjusted using the “Threshold” function in ImageJ. The intensities of Lamin A/C and Lamin B were assessed through the “Measure” function, and the ensuing values were recorded. The ratio of Lamin A/C to Lamin B was calculated by dividing the measured intensity value of Lamin A/C by the corresponding value of Lamin B. This process was reiterated across multiple images or regions of interest to establish an average ratio of Lamin A/C to Lamin B.

### 4.11. Analysis of Nuclear Solidity

Nuclear solidity was assessed using ImageJ software. Fluorescent images featuring the cell nucleus (stained with DAPI) were converted into a binary format. The threshold was then adjusted to ensure accurate segmentation of the nucleus from the background. The “Fill Holes” function was used to achieve a complete representation of each nucleus, without any internal gaps. This function guarantees that any existing holes within the segmented nuclei are filled. Subsequently, the solidity of the nucleus was evaluated (selected under “shape descriptors” when setting measurement parameters). The corresponding results were recorded using the “Measure” function. This function calculates the solidity by determining the ratio of the area of the nucleus to the area of its convex hull (the smallest convex set that contains the nucleus). This process provides a measure of the nuclear integrity and shape consistency.

### 4.12. Image Processing Using Super-Resolution Radial Fluctuations (SRRF)

Super-resolution images were reconstructed using the NanoJ-Super Resolution Radial Fluctuations (SRRF) (Henriques Laboratory, Lisbon, Portugal) plugin for ImageJ [69,70,71]. Initially, the focus of the confocal microscope was adjusted to locate the region of interest. The images were then captured in the same region with an exposure time of 1 s, with a total of 100 frames for each stack. Following capture, the raw image series were processed using the integrated algorithms of the NanoJ-SRRF plugin. These algorithms served to minimize residual artifacts and background noise, thereby enhancing image quality. To achieve better resolution of actin fibers, the ring radius parameter was adjusted to 0.5, and the radiality magnification was set to 5. This allowed for a more detailed and precise visualization of the actin fibers in the reconstructed super-resolution images.

### 4.13. FRET Imaging

For the E-cadherin FRET analysis, p-35 20 mm glass-bottom dishes (Celvis) were coated with 20 μg/mL of fibronectin (FN) and allowed to stand for 15–20 min at room temperature. An adenovirus was used to express E-cadherin TSmod (previously developed as described in [72] and was generated by the VCU macromolecule core using the pADEasy system. The E-cadherin TSmod was expressed in the doxycycline-inducible MDCK cells and MDCK wild-type cells. The transfected cells were grown in the FN-coated dishes for 24 h until 80% confluency was reached, following which, the cells were induced for the mutation with 10 ng/mL of doxycycline for a time period of 24 h, and FRET measurements were taken against the non-induced control. For the Nesprin-2 FRET experiments, p-35 20 mm glass-bottom dishes (Celvis) were coated with 20 μg/mL of fibronectin (FN) and allowed to stand for 15–20 min at room temperature. A Nesprin-2G TSmod MDCK stable cell line was developed using G418 selection [73]. These cells were then infected using a constitutively active lentivirus to express p60AmotL2. FRET measurements were taken for the MDCK p60AmotL2 cells stably expressing the Nesprin-2G FRET sensor and compared against the Nesprin tension sensor module that served as the control. The FRET images were acquired using a plan-apochromat 40× water immersion NA 1.1 objective lens on an inverted Zeiss LSM 710 laser scanning microscope (Oberkochen, Germany) at the 458 nm wavelength from an argon laser source. For FRET analysis, the donor and acceptor channels were obtained via spectral unmixing. The acquired intensity images were processed and analyzed with the help of a custom Python code, as previously described in [73]. For each dataset, at least 10 images were obtained and were masked manually on Image J (Fiji). The FRET index images were obtained by taking the ratio of the acceptor fluorophore channel to the donor fluorophore channel, which was then multiplied with the binary image masks outlining the cell–cell junctions or nuclear envelope to measure only the FRET pixels in the region of interest.

### 4.14. Micropipette Force Probe (MFP)

#### 4.14.1. Preparation of Microbeads and Micropipette

The amino microbeads (Dynabeads, product no. M270, Thermo Fisher Scientific) were first treated with 1% glutaraldehyde for 1 h at room temperature. After being washed with 1X PBS three times, the beads were incubated in a 10 µg mL^−1^ E-cadherin (product no. 10204-H08H, Sino Biological, Beijing, China) solution overnight at 4 °C with agitation to avoid aggregation. Then, the beads were washed and stored in 1X PBS at 4 °C with agitation for use. The micropipettes were made from thin-wall glass filament (TW100F-6, World Precision Instruments, Sarasota, FL, USA) using a micropipette puller (P97, Sutter Instrument, Novato, CA, USA). Then, the micropipettes were cut by a heated platinum wire to obtain a smooth opening tip with a diameter of approximately 2 µm, suitable for capturing 3 µm microbeads.

#### 4.14.2. Adhesion Force Measurement

The cells were plated in a homemade chamber that was coated with 10 µg/mL fibronectin. For the overexpression of p60AmotL2 and p100AmotL2, 10 ng/mL doxycycline was added 2 h after cell attachment. After 24 h of incubation, the E-cadherin coated microbeads were added into the chamber. The micropipette mounted on an MP-285 micromanipulator was gently inserted into the chamber at a small angle and captured an E-cadherin coated bead through liquid aspiration pressure. The bead was then moved down to touch the cell surface with a contact force of approximately 500 pN (normally ~6 µm bending distance of the micropipette on the Z-direction) for 3 min. The cell was subsequently moved away from the micropipette by controlling the microscopy stage at a speed of 1.5 µm s^−1^, resulting in the micropipette bending until the adhesion interface rupture (Figure 7B). The micropipette experiments were recorded and analyzed using ImageJ to determine the micropipette deflection distance (Δx). The amount of force applied to the adhesion interface could then be evaluated from the micropipette deflection distance (Δx) based on the pre-calibrated bending stiffness (k) of the micropipette (*F* = *k*Δ*x*).

#### 4.14.3. Micropipette Calibration

Each micropipette used in our experiment was calibrated using a standard micro glass rod with a known bending stiffness (ks = 21.09 ± 4.22 pN μm^−1^) following a previously reported method [74]. Briefly, the micropipette tip was pressed by the glass rod tip with a step of d. The force applied to the contact interface can be calculated by the micropipette deflection (Δx): *F* = *k*Δ*x* = *ks*(*d* − Δ*x*). This means the bending stiffness of the micropipette is: *k* = *ks*(*d* − Δ*x*)/Δ*x*. The bending stiffness of the standard micro glass rod (ks) was calibrated by a transverse magnetic tweezer, where a micropipette capturing a magnetic bead was bent by the transverse magnetic tweezer with different forces [75].

#### 4.14.4. Nucleus Displacement

In addition to plating cells on a fibronectin-coated glass surface (stiff substrate), PDMS was also introduced as a soft substrate to reduce the effect of focal adhesion on nucleus displacement. A 1:50 PDMS mixture was spin-coated on the glass surface with a 50 µm thickness and cured at 80 °C, resulting in a Young’s modulus of approximately 10 kPa [76]. The PDMS was then incubated with fibronectin and used for plating MDCK p60AmotL2 cells. Hoechst (1000 dilution, Thermo Fisher Scientific) was added into the chamber before the micropipette experiment to monitor nucleus displacement or size change during the E-cadherin-coated bead pulling. The recorded fluorescence images were analyzed using ImageJ to obtain the nucleus size change in the parallel and vertical directions relative to the force direction. Then, the nucleus size change ratios were calculated by comparing the initial size before pulling.

### 4.15. Atomic Force Microscope (AFM)

The atomic force microscope used for the elasticity measurements was an XE120 model (Park Systems, Suwon, Republic of Korea) working in a force spectroscopy mode. The AFM is equipped with a liquid cell sitting on an XY piezoscanner with a range of 100 × 100 μm^2^. The approach and retraction of the AFM probe are realized using a separate piezoscanner with a Z-range of 25 μm. To estimate the elastic properties of the investigated samples, force curves were recorded in 36 different positions on a cell within a scan area of 10 μm × 10 μm. Triplicate measurements were carried out in liquid conditions for cells. The curves were acquired at an approach speed of 8 μm/s with the ORC8 cantilevers (a nominal spring constant of 0.1 N/m). The difference between force curves recorded on stiff glass and soft samples enables the determination of the force vs. indentation curves, which is the basis for the calculations of the elastic modulus. The elastic modulus (i.e., the Young’s modulus) was determined from the Hertz–Sneddon model, assuming that the indenting AFM probe had a conical shape [77].

### 4.16. Transwell Migration Assay

Transwell inserts featuring pore sizes of 8 μm (product no. 3422, Corning Inc., Corning, NY, USA) and 3 μm (product no. 3399, Corning Inc.) were employed for this procedure. The cells, subject to doxycycline induction, were dissociated via trypsinization, suspended in serum-deprived DMEM medium and then loaded onto the upper chamber of the transwell inserts. These inserts were subsequently positioned on the corresponding wells of a 24-well plate. The lower chambers were filled with DMEM medium supplemented with 5% FBS and the plates were incubated at 37 °C for a period of 4 h. Following incubation, the membranes from the transwell inserts were excised and mounted onto chamber slides. Cells that had migrated onto the transwell membranes were stained with phalloidin (to highlight F-actin) and DAPI (a nuclear stain) and then enumerated to assess migration rates. This provided insights into the mobility and invasive potential of the cells.

### 4.17. Statistics

The sample size, statistical test used and level of significance are stated in connection with each occurrence.

## Data Availability

No new data were created or analyzed in this study. Data sharing is not applicable to this article.

## Supplementary Materials

The following supporting information can be downloaded at: https://www.mdpi.com/article/10.3390/cells12131682/s1, Figure S1: p60AmotL2 expression phenocopies p100AmotL2 depletion; Figure S2: MDCK control cells grown in 3-D collagen in the presence or absence of Dox. Related to Figure 3; Figure S3: Adenoviral expression of p60AmotL2 promotes invasion of MDCK cells in 3-D collagen; Figure S4: p60AmotL2 promotes migration through 3 μm trans-well filters; Figure S5: Full length blots related to Figure 1B; Figure S6: Related to Figure 1C and D; Figure S7:Full length blots related to Figure 6E; Video S1: Movie related to Figure 3A top panel; Video S2: Movie related to Figure 3A bottom panel; Video S3: Movie related to Figure 3D top panel; Video S4: Movie related to Figure 3D bottom panel.
