# Supplementary material for: Modulation of E-Cadherin Function through the AmotL2 Isoforms Promotes Ameboid Cell Invasion
Source: Cells. 2023 Jun 21;12(13):1682. doi: 10.3390/cells12131682 (PMC10340588; doi:10.3390/cells12131682)
Supplement: Supplementary file 1 [file cells-12-01682-s001.zip › Cells_Holmgren_Suppl. Figs. 230602.pdf]

Modulation of E-cadherin function through the AmotL2 isoforms promotes ameboid cell invasion

Supplemental Figures:

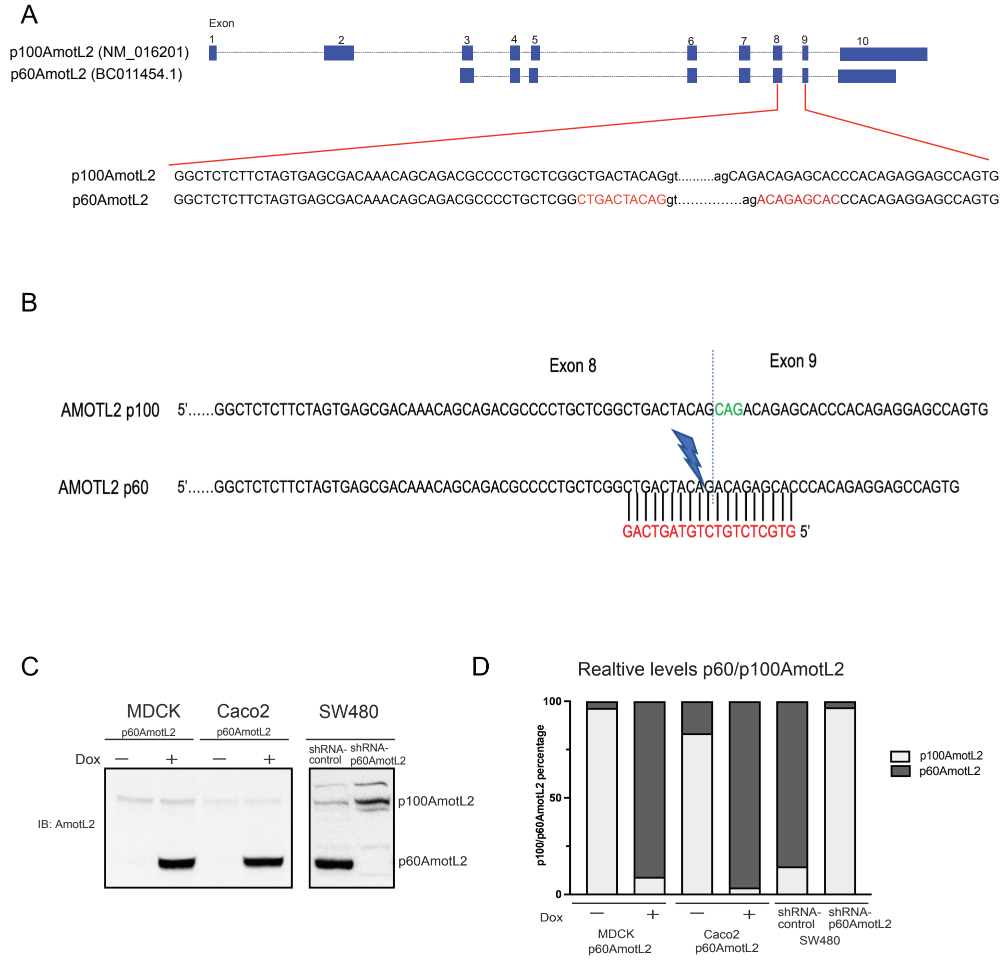

Figure S1. p60AmotL2 expression phenocopies p100AmotL2 depletion. Related to Figure 1. A) An illustration of transcripts encoding p100 and p60 of AMOTL2. Exons are shown as blue boxes and introns as dotted lines. The target sequence of AMOTL2 p60 is highlighted in red. The small letters “gt” and “ag” refer to splicing donor and acceptor sites, respectively. B) Splicing junction between exon 8 and 9 for both AMOTL2 p100 and p60. Three additional

nucleotides “CAG” (in green) are present in the AMOTL2 p100, compared to the p60. The sequence of the guide siRNA targeting AMOTL2 p60 (in red) is perfectly complementary to the splicing junction between exon 8 and 9 of the AMOTL2 p60. The cleavage site is indicated by flash sign.

C) Western blot for the analysis of relative p100 and p60 AmotL2 levels. MDCK and Caco-2 cells were transfected with Dox-inducible p60AmotL2. Right panel shows expression of endogenous p60AmotL2 in SW480 cells. Note the specific depletion of the p60 isoform as indicated. D) Bar diagram depicts relative levels of the two AmotL2 isoforms.

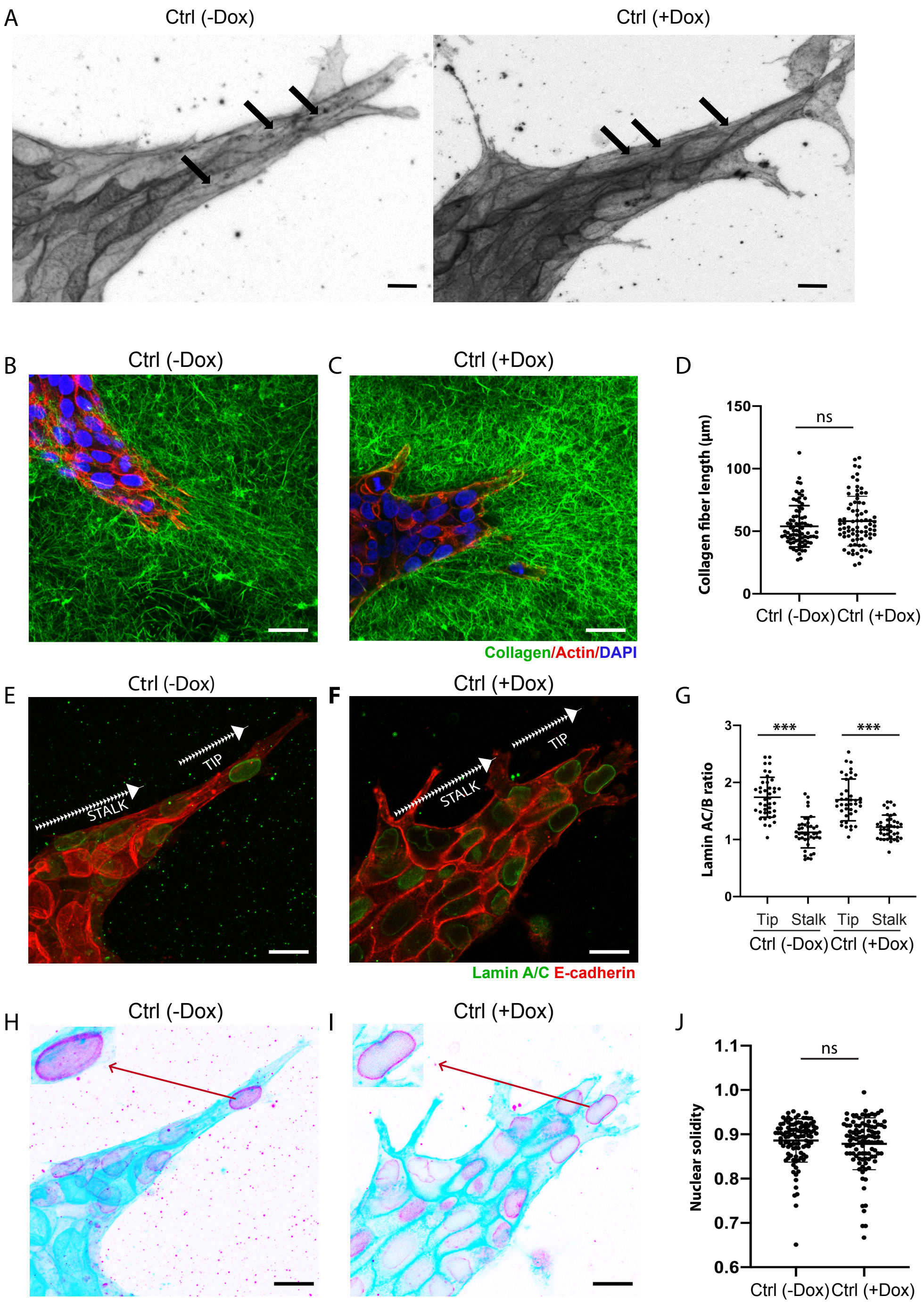

Figure S2. MDCK control cells grown in 3-D collagen in the presence or absence of Dox. Related to Figure 3. A) Inverted figures in black and white showing actin filaments in tube forming sprouts of MDCK control – or + Dox. Actin filaments were visualized by phalloidin staining. The addition of Dox did not affect actin filament formation during MDCK

tubulogenesis. Sixe bars=100 mm. B and C) Analysis of the interaction of MDCK cells with collagen fibers. Phalloidin is shown in red and collagen fibers were labeled with Oregon green. D) Dot plot diagram shows no significant effect of Dox on the length of collagen fibers (exemplified in B and C) ($n = 75$, Mann-Whitney U-test, n.s.). E and G) Dox treatment did not affect the Lamin A/C to Lamin B ratio ($n = 40$, Mann-Whitney U-test, ***$p < 0.001$). H and J) No effect of Dox on the solidity of MDCK cell nuclei during tubulogenesis ($n = 102$, Mann-Whitney U-test, n.s.). Data is represented as Mean +- SD and all experiments were performed at least 3 times with similar results. Size bars = 20 um.

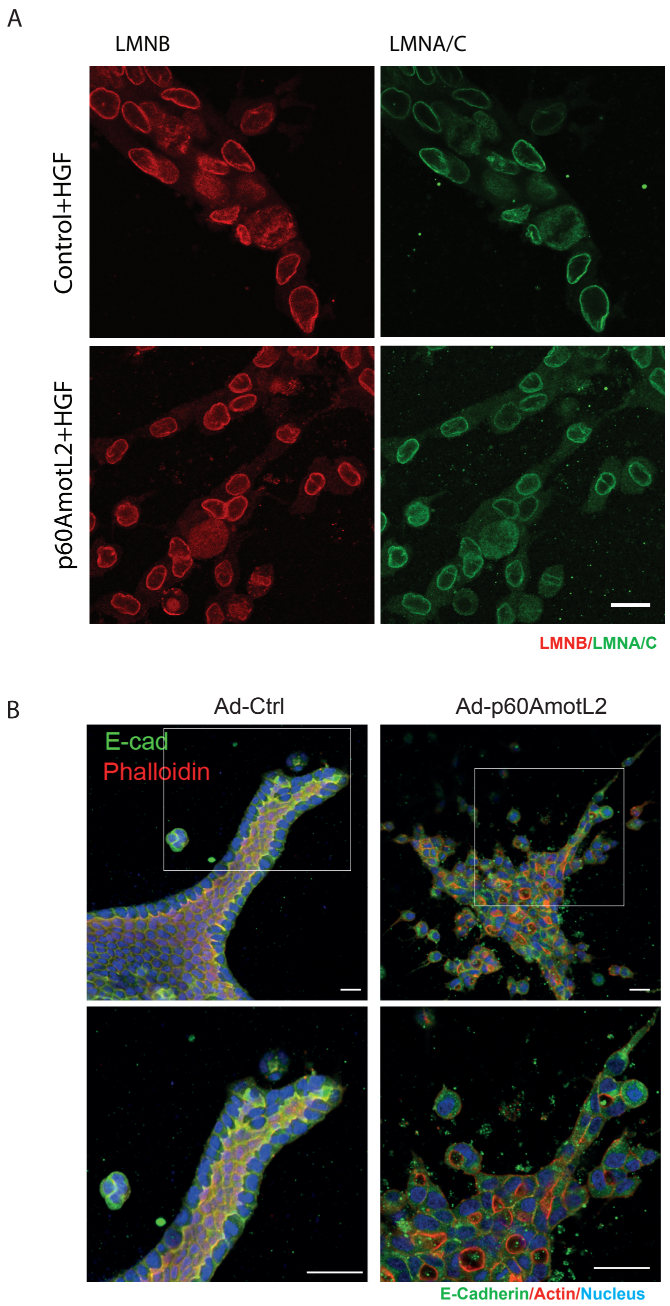

Figure S3. Adenoviral expression of p60AmotL2 promotes invasion of MDCK cells in 3-D collagen. MDCK cells expressing Ad-Ctrl or Ad-p60AmotL2 adenovirus were grown in 3-D collagen and induced with HGF. Collagen gels were fixed with PFA and immunostained using antibodies against E-cadherin and actin was visualized using phalloidin staining. Note the loss

of cell contacts in the p60AmotL2 expressing cells. Size bars = 20μm. All experiments were performed three times with similar results.

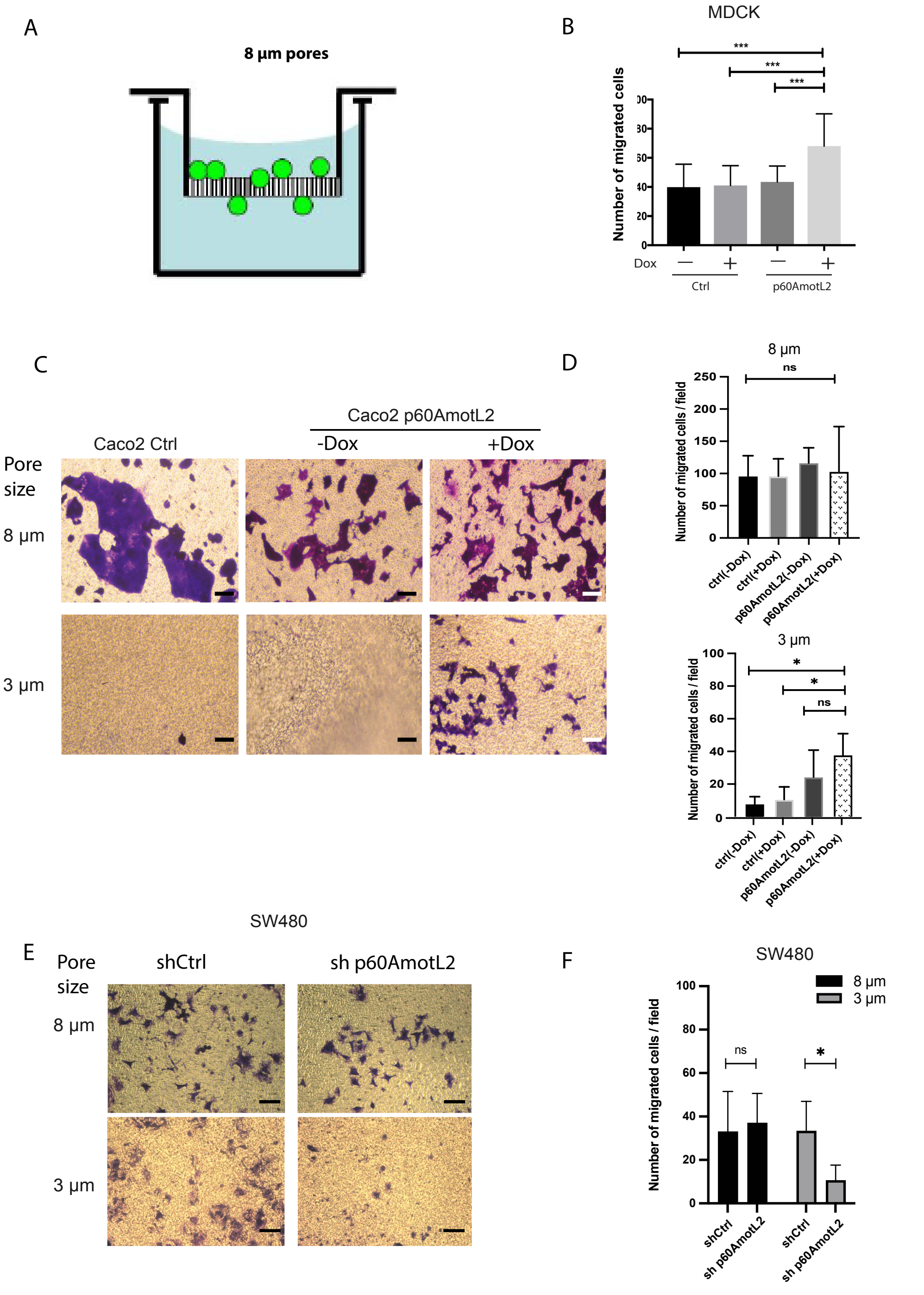

Figure S4. p60AmotL2 promotes migration through 3um trans-well filters. Related to Figure 9. A) Scheme of cells migrating through 8µm pores. B) Bar graph representing the MDCK cells migrating through 8µm pores (Mann-Whitney U-test, ***p<0.001). C and E) Brightfield images of migrated Caco-2 and SW480 cells on 8 or 3 µm transwell filters stained with Crystal violet (3 replicates). Scale bars = 20 µm. D) Bar diagram showing quantification of cells migrated through the 3 or 8 µm pore transwell filter in each group (Mann-Whitney U-test, *p<0.1). E) Crystal violet staining of SW480 cells migrating through 8 and 3mm pores in a trans well assay. F) Bar diagrams showing that depletion of p60AmotL2 inhibited SW480 cells ability to migrate through the 3 µm pores (Mann-Whitney U-test, *p<0.1). *P<0.05, **P<0.01, ***P<0.001.

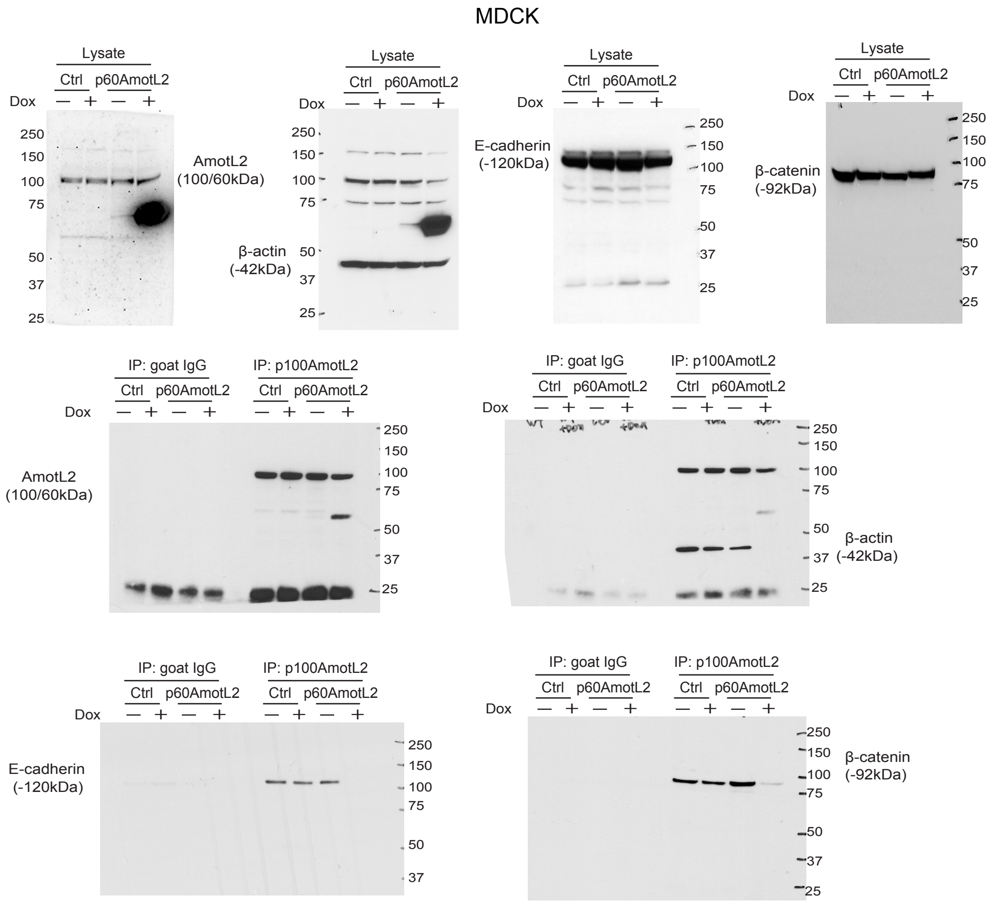

Full length blots related to Figure 1B.

Figure S5. Full length blots related to Figure 1B.

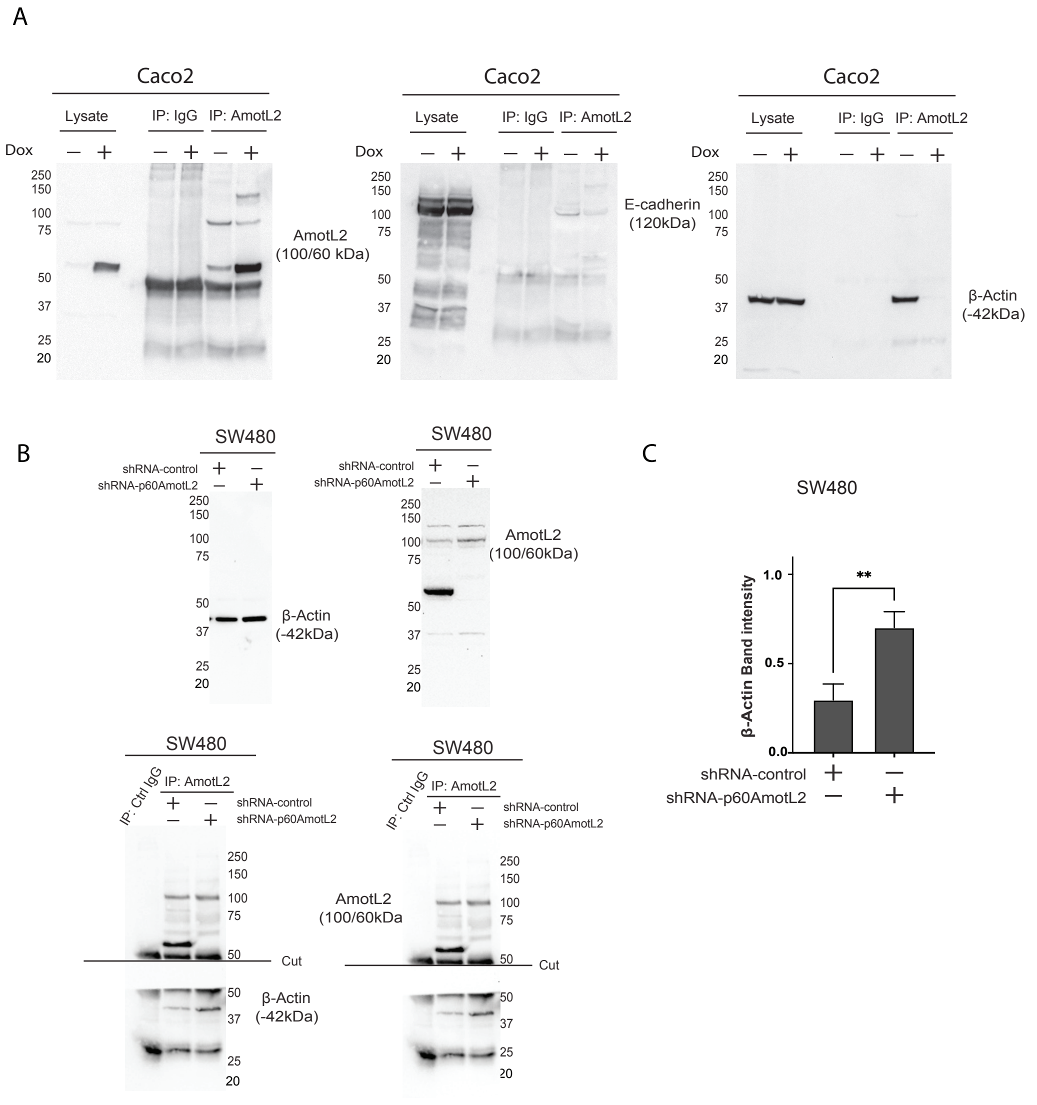

Related to Figure 1C and D.

A).Full western blots presented in figure 1C. B).Full western blots presented in figure 1D.C).Quantification of β-Actin intensity of SW480 ShRNA-Ctrl and p60AmotL2 KD cells.

Figure S6. Related to Figure 1C and D. A) Full western blots presented in figure 1C. B) Full western blots presented in figure 1D. C) Quantification of β-Actin intensity of SW480 ShRNA-Ctrl and p60AmotL2 KD cells (n = 3, Mann-Whitney U-test, $^{**}p<0.01$).

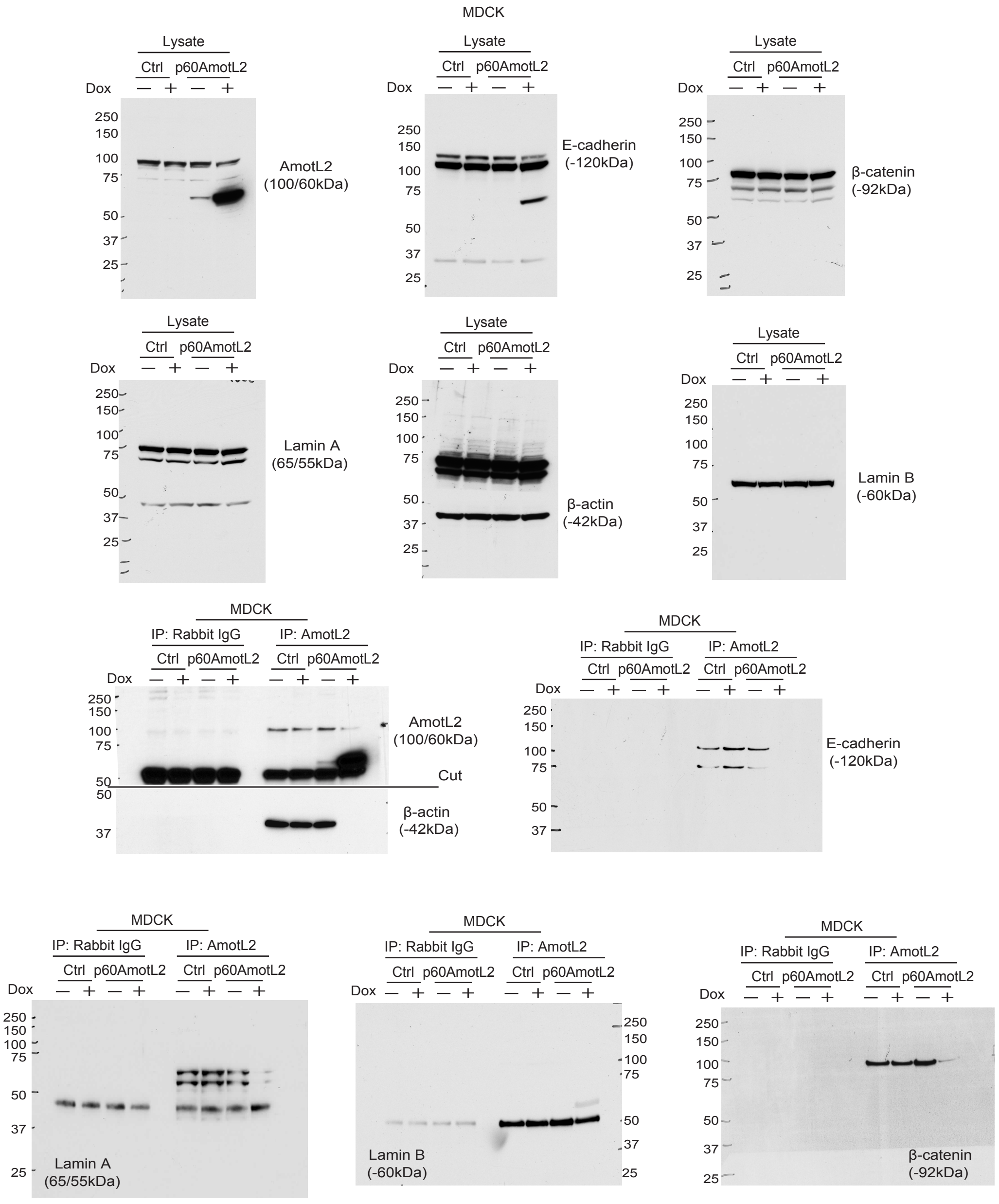

Full length blots related to Figure 6E.

Figure S7. Full length blots related to Figure 6E.
